# Association of Genetic Variants Linked to Late-Onset Alzheimer Disease With Cognitive Test Performance by Midlife

**DOI:** 10.1001/jamanetworkopen.2022.5491

**Published:** 2022-04-04

**Authors:** Scott C. Zimmerman, Willa D. Brenowitz, Camilla Calmasini, Sarah F. Ackley, Rebecca E. Graff, Stephen B. Asiimwe, Adam M. Staffaroni, Thomas J. Hoffmann, M. Maria Glymour

**Affiliations:** 1 Department of Epidemiology and Biostatistics, University of California, San Francisco; 2 Department of Psychiatry and Behavioral Sciences, University of California, San Francisco; 3 Weill Institute for Neurosciences, Department of Neurology, Memory and Aging Center, University of California, San Francisco; 4 Institute for Human Genetics, University of California, San Francisco

## Abstract

**Importance:**

Identifying the youngest age when Alzheimer disease (AD) influences cognition and the earliest affected cognitive domains will improve understanding of the natural history of AD and approaches to early diagnosis.

**Objective:**

To evaluate the age at which cognitive differences between individuals with higher compared with lower genetic risk of AD are first apparent and which cognitive assessments show the earliest difference.

**Design, Setting, and Participants:**

This cross-sectional study used data from UK Biobank participants of European genetic ancestry, aged 40 years or older, who contributed genotypic and cognitive test data from January 1, 2006, to December 31, 2015. Data analysis was performed from March 10, 2020, to January 4, 2022.

**Exposure:**

The AD genetic risk score (GRS), which is a weighted sum of 23 single-nucleotide variations.

**Main Outcomes and Measures:**

Seven cognitive tests were administered via touchscreen at in-person visits or online. Cognitive domains assessed included fluid intelligence, episodic memory, processing speed, executive functioning, and prospective memory. Multiple cognitive measures were derived from some tests, yielding 32 separate measures. Interactions between age and AD-GRS for each of the 32 cognitive measures were tested with linear regression using a Bonferroni-corrected *P* value threshold. For cognitive measures with significant evidence of age by AD-GRS interaction, the youngest age of interaction was assessed with new regression models, with nonlinear specification of age terms. Models with youngest age of interaction from 40 to 70 years, in 1-year increments, were compared, and the best-fitting model for each cognitive measure was chosen. Results across cognitive measures were compared to determine which cognitive indicators showed earliest AD-related change.

**Results:**

A total of 405 050 participants (mean [SD] age, 57.1 [7.9] years; 54.1% female) were included. Sample sizes differed across cognitive tests (from 12 455 to 404 682 participants). The AD-GRS significantly modified the association with age on 13 measures derived from the pairs matching (range in difference in mean cognition per decade increase in age for 1-SD higher AD-GRS, 2.5%-11.5%), symbol digit substitution (range in difference in mean cognition per decade increase in age for 1-SD higher AD-GRS, 2.0%-5.8%), and numeric memory tests (difference in mean cognition per decade increase in age for 1-SD higher AD-GRS, 8.8%) (*P* = 1.56 × 10^−3^). Best-fitting models suggested that cognitive scores of individuals with a high vs low AD-GRS began to diverge by 56 years of age for all 13 measures and by 47 years of age for 9 measures.

**Conclusions and Relevance:**

In this cross-sectional study, by early midlife, subtle differences in memory and attention were detectable among individuals with higher genetic risk of AD.

## Key Points

**Question:**

At what age do individuals with higher genetic risk of Alzheimer disease first show cognitive differences from individuals with lower genetic risk, and which of 32 cognitive measures show the earliest difference?

**Findings:**

In this cross-sectional study of 405 050 individuals, higher genetic risk of Alzheimer disease significantly modified the association of age with 13 of 32 cognitive measures. Best-fitting models suggested that higher genetic risk of Alzheimer disease was associated with changes in cognitive scores of individuals older than 56 years for all 13 measures and older than 47 years for 9 measures.

**Meaning:**

These findings suggest that by early midlife, subtle differences in cognitive measures may emerge among individuals with higher genetic risk of Alzheimer disease.

## Teaser

This cross-sectional study of UK Biobank participants older than 40 years evaluates the youngest age at which the genetic risk scores for Alzheimer disease are associated with cognitive outcomes.

## Introduction

A diagnosis of Alzheimer disease (AD) is preceded by a decades-long process of accumulating cerebral pathology.^1,2^ However, neither the precise age when symptoms of disease-related pathology begin nor the earliest symptomatic manifestations have been established. Identification of the earliest indicators of AD would improve understanding of the course of disease development. Identification of cognitive domains most sensitive to early changes would help guide effective screening, prevention, and treatment.

Longitudinal study designs that measure midlife cognition and late-life AD are impractical for identifying the timing and cognitive domains of the earliest AD manifestations. Such studies would require decades of follow-up and could not distinguish cognitive reserve from early disease-related changes. Innovations using genetic information offer more practical study designs.^3^ With the use of a genetic risk score (GRS) that is associated with AD development in late life, it is possible to detect early symptoms of AD in existing midlife cohorts.^4,5,6^ The AD-GRS is determined before early-life phenotypes, but associations between the AD-GRS and AD symptoms emerge as individuals age. The earliest AD symptoms are subtle, requiring a large sample for detection. Most previous studies^7,8,9,10,11,12,13,14^ of genetics and cognition used populations older than 65 years. Some studies^8^ examining associations of genetics with overall cognition in middle-aged to older adults have found that effects are stronger after 65 years of age. Few large-scale studies of middle-aged to older adults have comprehensively examined interactions between age and AD genetic risk with multiple cognitive measures.

Although episodic memory changes are generally considered leading indicators of AD,^15,16,17,18,19,20^ subtle changes in other domains, such as semantic memory, processing speed, and executive functioning, may occur at the same age or earlier.^20,21,22,23^ The potential for symptoms to manifest in any of multiple cognitive domains can be evaluated in parallel using a hypothesis-free approach to rapidly screen numerous possible indicators of disease based on phenotypes associated with a GRS.^24,25^ We adapt this method to evaluate potential early cognitive indicators of AD.

Because the hallmark of AD is age-related emergence of cognitive deficits,^26^ we applied an hypothesis-free method to identify cognitive domains differentially associated with the combination of aging and an AD-GRS. Considering 32 cognitive function indicators covering heterogeneous cognitive domains, we evaluated the youngest age at which the AD-GRS was associated with cognitive outcomes and which cognitive assessments showed the earliest changes. By estimating models in the large UK Biobank study, we had excellent power to detect subtle associations.

## Methods

### Study Setting and Participants

The UK Biobank is an ongoing cohort study, described in detail elsewhere.^27^ More than 500 000 individuals aged 40 to 69 years enrolled from January 1, 2006, to December 31, 2010, providing biological samples and survey responses. Online or in-person cognitive assessments were fielded for some or all of the study participants. Given the challenge of participation in the UK Biobank study, the prevalence of mild cognitive impairment and dementia is low.^28,29^ Ethical approval for UK Biobank data collection was obtained from the National Health Service National Research Ethics Service; all participants provided written informed consent. Analyses for the current cross-sectional study were based on fully deidentified data with no access to identifiers and therefore deemed not human subjects research by the University of California, San Francisco Institutional Review Board. This study follows the Strengthening the Reporting of Observational Studies in Epidemiology (STROBE) reporting guideline for cross-sectional studies.^30^

From 497 087 UK Biobank participants 40 years or older at baseline, we excluded participants with 1 or more of the following: missing genetic information (n = 15 210 [3.1%]), non-European genetic ancestry (n = 78 494 [15.8%]), or no completed cognitive tests (n = 946 [0.002%]). Our final eligible sample included 405 050 participants. Cognitive assessments were not all conducted for all respondents, and our analytic sample ranged from 12 455 to 404 682 participants across the cognitive tests (Table 1), with data collected from January 1, 2006, to December 31, 2015. Data analysis was performed from March 10, 2020, to January 4, 2022.

**Table 1.** Sample Composition

Variable	No. of participants	Age, mean (SD), y^a^	Female, %	AD-GRS, mean (SD)	AD-GRS range
**Fluid intelligence (logic and reasoning)**					
No. correct in person	128 550	57.5 (7.8)	53.8	0.012 (0.996)	0-13
No. attempted in person	128 550	57.5 (7.8)	53.8	0.012 (0.996)	1-13
No. correct online	102 873	62.8 (7.5)	55.4	0.002 (0.989)	0-14
**Numeric memory (short-term memory capacity and attention)**					
No. correct in person	41 811	57.2 (7.9)	54.2	0.011 (0.996)	2-12
No. correct online	92 625	62.6 (7.5)	55.4	0.001 (0.989)	2-11
**Pairs matching (short-term memory and attention)**					
No. correct in person (round 1)	404 682	57.1 (7.9)	54.1	0.019 (1.001)	0-8
No. correct in person (round 2)	404 682	57.1 (7.9)	54.1	0.019 (1.001)	0-6
No. incorrect in person (round 1)	404 682	57.1 (7.9)	54.1	0.019 (1.001)	0-146
No. incorrect in person (round 2)	404 682	57.1 (7.9)	54.1	0.019 (1.001)	0-146
Time to complete in person (round 1)	396 627	57 (7.9)	54.1	0.018 (1)	40-13 751
Time to complete in person (round 2)	395 763	57 (7.9)	54.2	0.018 (1)	91-13 751
No. correct online (round 1)	98 753	62.8 (7.5)	55.5	0.003 (0.99)	0-8
No. incorrect online (round 1)	98 753	62.8 (7.5)	55.5	0.003 (0.99)	0-50
No. incorrect online (round 2)	12 567	60.4 (7.6)	55	−0.003 (0.989)	0-33
Time to complete online (round 1)	95 178	62.6 (7.5)	55.7	0.001 (0.988)	2195-99 988
Time to complete online (round 2)	12 455	60.3 (7.6)	54.9	−0.004 (0.988)	10851-99 988
**Prospective memory**					
First attempt correct in person	131 255	57.5 (7.8)	53.8	0.013 (0.997)	0-1
First or second attempt correct in person	131 255	57.5 (7.8)	53.8	0.013 (0.997)	0-1
**Reaction time **					
Mean time	402 528	57.1 (7.9)	54.1	0.018 (1.001)	63-2000
**Symbol digit substitution (processing speed)**					
No. attempted in person	13 986	63.8 (7.3)	50.9	0.006 (0.984)	1-105
No. correct in person	13 986	63.8 (7.3)	50.9	0.006 (0.984)	0-37
No. correct online	98 692	62.8 (7.5)	55.4	0.002 (0.99)	0-103
No. attempted online	98 692	62.8 (7.5)	55.4	0.002 (0.99)	1-109
Time to complete 10 substitutions online	75 997	62.6 (7.5)	54.8	−0.001 (0.985)	12 146-152 295
**Trail-making (processing speed and/or executive functioning)**					
Time to complete numeric trail in person	13 958	63.8 (7.3)	50.8	0.007 (0.984)	94-2557
No. of errors in numeric trail in person	14 093	63.8 (7.3)	50.9	0.007 (0.985)	0-147
Time to complete alphanumeric trail in person	13 638	63.7 (7.3)	50.8	0.005 (0.984)	160-5768
No. of errors in alphanumeric trail in person	14 093	63.8 (7.3)	50.9	0.007 (0.985)	0-165
Time to complete numeric trail online	86 861	62.5 (7.5)	54.5	−0.002 (0.987)	13.666-733.97
Time to complete alphanumeric trail online	86 859	62.5 (7.5)	54.5	−0.002 (0.987)	20.556-746.531
No. of errors in numeric trail online	22 029	63.2 (7.5)	59.7	0.019 (0.998)	1-536
No. of errors in alphanumeric trail online	35 462	63.6 (7.4)	57.2	0.007 (0.997)	1-320
**Full sample**					
Total	404 050	57.1 (7.9)	54.1	0.029 (1)	NA

Abbreviations: AD, Alzheimer disease; GRS, genetic risk score; NA, not applicable.

^a^ For each cognitive test, the age represents the age at testing. For the full sample, age represents the age at recruitment.

### Genotyping and AD Genetic Risk Scores

UK Biobank samples were genotyped in batches of approximately 4700 with 2 assays (UK BiLEVE array and UK Biobank Axiom array) using genotyping and quality control methods detailed elsewhere.^31,32^ We calculated AD-GRSs using single-nucleotide variations (SNVs) identified in a meta-analysis of genome-wide association studies of AD.^33^ The AD-GRS is a weighted sum of 23 SNVs that has been validated as being associated with dementia outcomes in prior work.^4,5,6,34^ For the goal of this study, it was essential that the AD-GRS is associated with dementia. Exploratory evaluations demonstrated that the AD-GRS is associated with an AD diagnosis in the UK Biobank (eAppendix 1 and eTables 1 and 2 in the Supplement). In quality control checks, we found that the AD-GRS was more strongly associated with cognition based on verbal reasoning than a newer GRS that included additional loci from a 2019 International Genomics of Alzheimer Project meta-analysis.^35^ Higher AD-GRS scores correspond to higher risk of developing AD. For analysis, we standardized the AD-GRS by centering at the full sample’s mean and dividing by its SD

Our AD-GRS included weights for apolipoprotein E (*APOE*) ε4 alleles. In sensitivity analyses, we used an alternative AD-GRS calculated without SNVs from the *APOE* region. This alternative AD-GRS included 21 SNVs (eAppendix 2 and eTable 3 in the Supplement). In addition, we used the count of *APOE* ε4 alleles in place of the AD-GRS measure to assess the impact of *APOE*. All AD-GRSs were created using PLINK, version 1.9.^36,37^

### Cognitive Measures (Phenotypes)

Cognitive measurements were conducted in person at UK Biobank assessment centers and through online follow-up. We considered only cognitive measures available for at least 10 000 individuals. Domains assessed using 7 instruments included fluid intelligence, episodic memory, processing speed, executive functioning, and prospective memory. Assessments as fielded in the UK Biobank are detailed in eAppendix 3 in the Supplement. Multiple measures were derived from some tests, including total score, component or round scores, completion status for the entire test or rounds of the test, and duration to complete a test or its components or rounds (eAppendix 2 and eTable 4 in the Supplement). This process resulted in 32 variables, all of which were coded such that larger positive values correspond to better performance.

When measures were obtained at multiple visits, we used the earliest available measure for each test to maximize sample sizes. Online and in-person versions of the same instrument were treated as distinct phenotypes because, even when measures tap the same underlying construct, 1 mode of administration may be more sensitive to early changes. Some participants completed both the in-person and online versions of an instrument.

### Covariates

In addition to phenotype and GRS data, our analyses considered age at cognitive assessment, self-reported sex, genotyping assay (a binary indicator for whether the UK BiLEVE or UK Biobank Axiom array was used), assessment center (when applicable), practice effect (online assessments only; an indicator of whether the participant previously completed the assessment center version of the test0), and 10 genetic ancestry principal components provided by the UK Biobank to account for population stratification in the sample.

### Statistical Analysis

Our primary interests were to identify which cognitive assessments show population differences by AD-GRS at the youngest age and estimate the youngest age of differences in those outcomes. We first estimated regression models with the interaction of age and the AD-GRS for each of the 32 cognitive assessments to select a smaller set of cognitive phenotypes for more detailed evaluation. We fit models of cognitive score (*Y*) as a function of age, *z*-scored AD-GRS (ADGRS*_z_*), their interaction, and covariates *W_i_* using linear regression (identity link) for continuous or ordinal phenotype variables and logistic regression (logit link) for binary phenotype variables: *Y* ∼ Link (*b*_0_ + *b*_1_ Age + *b*_2_ ADGRS*_z_* + *b*_3_ ADGRS*_z_* × Age + ∑ *_i_b_i_W_i_*).

We estimated the association between age and each cognitive score for people with an average AD-GRS (ie, *b*_2_ [the age main term]), the difference in the age slope associated with a 1-SD higher AD-GRS (ie, *b*_3_ [the interaction of age and ADGRS*_z_*]), and the percentage increase in the rate of change with age associated with a 1-SD higher AD-GRS compared with someone with an average AD-GRS (ie, 100 × *b*_3_/*b*_2_). Our primary coefficient of interest, *b*_3_, provides an estimate of whether the association of the AD-GRS with the cognitive phenotype is stronger for people of older age. For each coefficient, we report point estimates and 95% CIs. Our code is available at GitHub.^38^

If the interaction of age and the AD-GRS was statistically significant for the cognitive measure at a Bonferroni-corrected threshold (*P* < .05/32 = 1.56 × 10^−3^), we next fit models to detect the age at which the AD-GRS was associated with changes in the cognitive measure using a novel application of a standard cross-validation technique for best-fit model choice. To detect the youngest age at which divergence occurred (ie, the age at which the population average cognitive measures begin to separate based on level of AD-GRS), a cognitive score (*Y*) was fit to a quadratic function of age (*t*, centered at 40 years), a threshold function to detect age of divergence, and covariates *W_i_: Y* ∼ Link (*b*_0_ + *b*_1_ *t* + *b*_2_ *t*^2^ + *b*_3_ ADGRS*_z_* × *I*(*t* > *t*_threshold_) × (*t* − *t* > *t*_threshold_)^3^ + ∑ *_i_b_i_W_i_*).

The threshold function was operationalized as an interaction between an indicator function *I*(*t*>*t*_threshold_) for age above an hypothesized minimum age of divergence (*t*_threshold_), age above the threshold cubed, and ADGRS*_z_*. This specification allowed modeled mean cognitive scores to smoothly diverge based on level of AD-GRS beginning at the specified threshold age. Below the threshold age, the association between AD-GRS and cognition (conditional on covariates) is constrained to zero so that the association between centered age and the outcome is governed by *b*_1_*t* + *b*_2_*t*^2^. Above the threshold, the cognitive score is allowed to diverge smoothly from *b*_1_*t* + *b*_2_*t*^2^ by adding a third-order term *b*_3_ADGRS*_z_* × (*t* − *t*_threshold_)^3^ (eAppendix 4 in the Supplement).

We evaluated alternative hypothesized ages of 40 to 70 years for the threshold. Models with different thresholds were compared based on the mean squared prediction error in a 10-fold cross-validation,^39^ and we selected the age threshold from the model with the minimum mean squared prediction error (eFigure 1 in the Supplement). Within a given covariate stratum, this approach indicates that, below the selected age threshold, the mean cognitive measures in the data are best represented by a single value at a given age. However, above the threshold value, the data are better represented by AD-GRS–specific mean cognitive measures at each age. The range of threshold values evaluated included the full range of participant ages in the sample for the cognitive measure. Thus, a selected threshold at the lowest tested value indicates that the data are better represented by AD-GRS–specific curves across ages, whereas a selected threshold at the upper end of the range indicates that the data are better represented by a single curve across ages regardless of AD-GRS. For demonstration, we then used this model to simulate and plot anticipated average cognitive measures across age at the median values of covariates, comparing higher (95th percentile) and lower (fifth percentile) AD-GRSs.

In sensitivity analyses, all models were replicated using the alternative AD-GRS that omitted SNVs in the *APOE* region. In addition, to test whether results were sensitive to our modeling assumptions, we repeated the analyses using a more rigid linear age term with a quadratic divergence term above the threshold and a more flexible cubic age term with a fourth-order divergence term above the threshold (eAppendix 5 in the Supplement).

## Results

A total of 405 050 participants (mean [SD] age, 57.1 [7.9] years; 54.1% female) were included (Table 1). Of the 32 cognitive outcomes evaluated using a Bonferroni-corrected *P* value threshold for statistical significance (*P* = 1.56 × 10^−3^), there was evidence that the AD-GRS modified the association of age with 13 measures derived from the pairs matching (range in difference in mean cognition per decade increase in age for 1-SD higher AD-GRS, 2.5%-11.5%), symbol digit substitution (range in difference in mean cognition per decade increase in age for 1-SD higher AD-GRS, 2.0%-5.8%), and numeric memory tests (difference in mean cognition per decade increase in age for 1-SD higher AD-GRS, 8.8%) (Table 2; eTable 5 in the Supplement). Among these measures, the difference in mean cognition per decade increase in age associated with a 1-SD higher AD-GRS was greatest for the number correct in round 1 of the online pairs matching task, with an 11.5% increase in age-related differences per 1-SD higher AD-GRS.

**Table 2.** Association of AD-GRS at 40 Years of Age and Modification of Age Slope by AD-GRS for Each Cognitive Assessment With a Statistically Significant Age by AD-GRS Interaction in Linear Regression Models

Variable	Age slope per decade for person with mean GRS (95% CI)^a^	Difference in age slope for person with 1-SD higher GRS (95% CI)^b^	Difference in mean cognition per decade increase in age for 1-SD higher AD-GRS, %^c^
**Pairs matching (short-term memory and attention)**			
No. correct online (round 1)	−0.124 (−0.132 to −0.115)	−0.014 (−0.023 to −0.006)	11.5
No. correct in person (round 1)	−0.091 (−0.095 to −0.087)	−0.007 (−0.011 to −0.003)	7.9
No. in person correct (round 2)	−0.093 (−0.097 to −0.089)	−0.009 (−0.013 to −0.005)	9.4
Time to complete in person (round 1)	−0.207 (−0.211 to −0.203)	−0.011 (−0.015 to −0.007)	5.3
No. incorrect in person (round 1)	−0.144 (−0.148 to −0.140)	−0.006 (−0.010 to −0.002)	4.4
Time to complete online (round 1)	−0.440 (−0.448 to −0.432)	−0.015 (−0.023 to −0.007)	3.5
Time to complete in person (round 2)	−0.312 (−0.315 to −0.308)	−0.008 (−0.012 to −0.004)	2.5
**Numeric memory (short-term memory and attention)**			
No. correct online	−0.180 (−0.189 to −0.172)	−0.016 (−0.025 to −0.007)	8.8
**Symbol digit substitution (processing speed)**			
No. correct in person	−0.599 (−0.620 to −0.579)	−0.035 (−0.056 to −0.014)	5.8
No. attempted in person	−0.621 (−0.642 to −0.601)	−0.036 (−0.056 to −0.015)	5.7
Time to complete 10 substitutions online	−0.521 (−0.531 to −0.513)	−0.024 (−0.033 to −0.015)	4.5
No. correct online	−0.588 (−0.594 to −0.579)	−0.014 (−0.022 to −0.007)	2.4
No. attempted online	−0.617 (−0.624 to −0.610)	−0.012 (−0.020 to −0.005)	2.0

Abbreviations: AD, Alzheimer disease; GRS, genetic risk score.

^a^ Coefficient on age term (*b*_2_) in eEquation 1 in eAppendix 6 in the Supplement.

^b^ Coefficient on AD-GRS*_z_* × age term (*b*_3_) in eEquation 1 in eAppendix 6 in the Supplement.

^c^ (100 × *b*_3_/*b*_2_) using the coefficients from eEquation 1 in eAppendix 6 in the Supplement.

**Figure 1.**
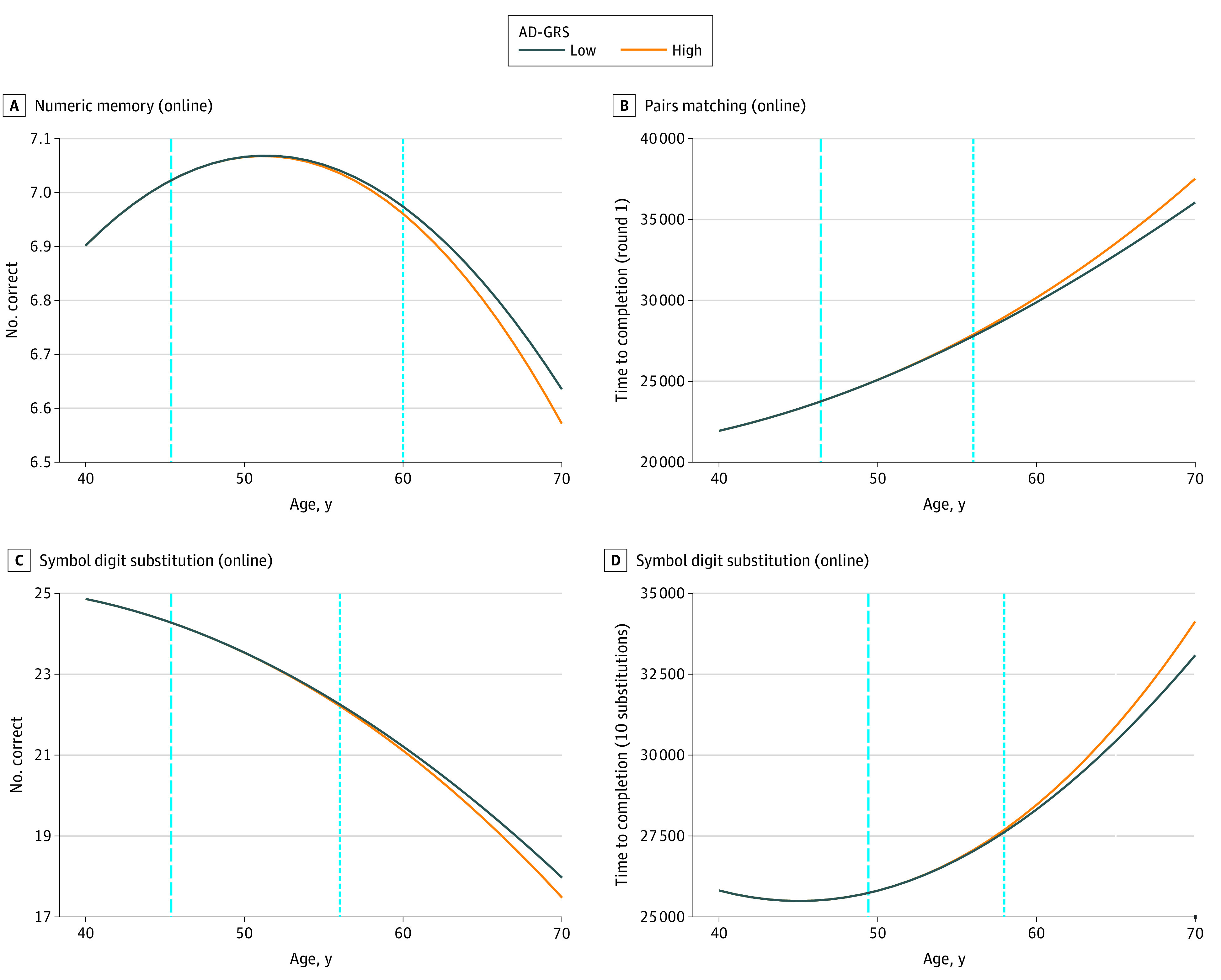
Divergence Model Results for 4 Cognitive Measures Plots of modeled mean cognition of 4 cognitive measures at higher (1.76) and lower (−1.22) *z*-scored Alzheimer disease genetic risk score (AD-GRS). Dashed vertical lines indicate threshold age at which the AD-GRS begins to be associated with the cognitive measure. At ages below the threshold, the AD-GRS does not interact with age to influence cognition. Dotted vertical lines indicate the earliest age at which the cognitive difference in the high and low AD-GRS groups are statistically detectable using a 1-sided, 2-sample *t* test (*P* ≤ .05). These curves were based on women with median values of the 10 principal components.

**Figure 2.**
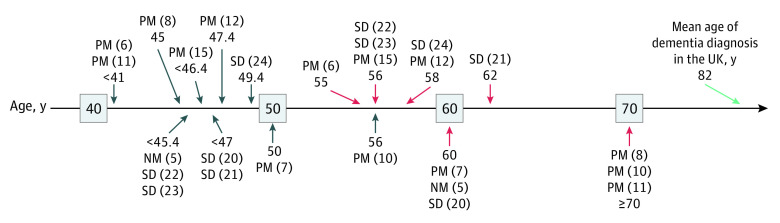
Age of Divergence of Cognitive Scores Associated With Higher Alzheimer Disease Genetic Risk Score (AD-GRS) and Age of Statistically Detectable Associations Illustration of age of divergence based on higher (95th percentile) and lower (fifth percentile) AD-GRS. The threshold age at which the AD-GRS begins to influence the population mean of the cognitive measure, as detected by the divergence model for that measure, are displayed with blue arrows. The age at which a *t* test is significantly different for high vs low AD-GRS for each cognitive measure is displayed with red arrows. The mean age of dementia diagnosis in the UK is displayed with a green arrow. NM indicates numeric memory; PM, pairs matching; and SD, symbol digit substitution.

From these 13 measures for which AD-GRS modified the association with age, we fit nonlinear models to estimate the youngest age of divergence in cognitive test performance between people with higher (95th percentile) vs lower (fifth percentile) AD-GRS (Figure 1 and Figure 2; eTable 6, and eFigures 2 and 3 in the Supplement). There was evidence of divergence at the youngest testable age for 8 of the 13 measures. The measures with the youngest observed age of divergence were for number correct in round 1 (age slope per decade for person with mean GRS in the corresponding linear model, −0.091; 95% CI, −0.095 to −0.087) and time to complete round 2 of the in-person pairs matching test (age slope per decade for person with mean GRS in the corresponding linear model, −0.312; 95% CI, −0.315 to −0.308). For those measures, the divergence occurred by age 40 years; because 40 years is the minimum enrollment age for the UK Biobank, no more specificity was possible. Best-fitting models suggested that cognitive scores of individuals with high vs low AD-GRSs began to diverge by 56 years of age for all 13 measures and by 47 years of age for 9 measures.

In sensitivity analyses, models using *APOE* ε4 allele count showed similar patterns to the main analysis (eAppendixes 6 and 7 and eTables 7-10 in the Supplement), and models using the AD-GRSs that omitted *APOE* ε4 had attenuated modification of age associations compared with the main analysis (eAppendix 8 and eTables 11 and 12 in the Supplement). Use of more flexible functional forms led to similar or earlier ages of divergence in most cases (eAppendix 8, eTables 13-15, and eFigures 4 and 5 in the Supplement).

## Discussion

Using a large UK Biobank sample, we found that people with higher AD genetic risk began to manifest subtle changes in 3 cognitive tests (pairs matching, symbol digit substitution, and numeric memory) by early middle age. Several measures from these tests suggested changes began before 45 years of age. Some measures appeared to diverge before the minimum age of study participants with available data, so we could not pinpoint the age of divergence. These results suggest early changes in cognitive domains of attention, short-term memory, and processing speed among participants at higher risk of developing clinical AD in later life based on genetic risk.

Sensitivity analyses excluding *APOE* showed slightly older ages of divergence, suggesting that, as expected, *APOE* ε4 allele carriers experience earlier cognitive decline. Our findings are consistent with prior work^1,15,16,17,18,21,40,41,42,43^ in early- and late-onset AD, suggesting that cognitive and physiologic changes associated with AD begin at least 15 years before diagnosis—the mean age of dementia diagnosis for White UK residents is 82 years.^44^ Our study advances understanding of the natural history of late-onset AD by showing that, in a generally healthy, community-dwelling sample, those at high genetic risk of developing AD in late life performed worse on several cognitive measures in midlife.

Several studies^7,8^ have examined AD-GRS or *APOE* in middle-aged to older adults. Although some studies^45,46,47^have found associations to be stronger in older adults, few studies^7,48^ have found significant interactions with age in adjusted analyses, including in a prior UK Biobank study^7^ of approximately 100 000 participants. Davies et al^8^ found a significant age association for *APOE* and general cognitive function after 65 years of age; however, outcomes were based on a meta-analysis^8^ of different cohorts generally of older ages (mean cohort aged 55-80 years). Our work adds to this literature in important ways. We used a large sample size, which allows for relatively stronger power to detect effects. We focused on identifying the youngest ages at which *APOE* or the AD-GRS was associated with differences in cognitive tests by 40 to 70 years of age. We examined 30 or more cognitive measures and prioritized those most strongly associated with AD-GRS. Finally, we compared the estimated associations between an AD-GRS with *APOE*, AD-GRS without *APOE*, and *APOE* ε4 allele alone.

Functional brain differences of *APOE* ε4 carriers compared with noncarriers have been observed among people in their 20s.^49,50^ A previous study^51^ found that reduced cognition in midlife or even earlier was associated with increased risk of AD. However, studies of midlife cognitive assessments and subsequent late-onset AD^52^ have not been able to distinguish whether cognitive assessments are associated with AD because they indicate cognitive reserve or because they are early manifestations of disease.^53^ For instance, cognitive test scores as early as 11 years of age may be associated with diagnosis of late-onset AD,^54^ but this association was more likely because early-life cognitive scores provide cognitive reserve or delay onset or diagnosis. Our study design avoided this ambiguity in interpretation because we focused on age-related differences rather than level of cognitive performance. Because we focused on detecting age-related differences (ie, the interaction of AD-GRS and age) and because the genetic measure of AD risk in late life is not influenced by early-life exposures, the measures that begin to diverge in midlife must be early symptoms of disease rather than indicators of cognitive reserve or variables that simply delay diagnosis of AD.

Episodic memory is often considered the earliest indicator of preclinical AD,^15,16,17,18,19,20^ but changes to category fluency, naming, executive functioning,^23^ and visuoconstruction abilities^55^ may also occur early,^40^ and multidomain approaches may be especially sensitive to preclinical AD.^15^ Our results are consistent with previous research^56,57^ that suggests that processing speed and short-term memory are particularly sensitive early indicators of cognitive decline. The findings of early cognitive changes are consistent with some prior biomarker research^41,58^ that documented changes in cerebral spinal fluid and neuroimaging markers by late midlife. The current study adds to these findings by showing early differences in processing speed and short-term memory or attention in those at high genetic risk for AD.

Our findings suggest that biological processes underlying AD may begin to exert clinical effects decades earlier than the age at which clinical trials commonly enroll patients with AD; for example, the Finnish Geriatric Intervention Study to Prevent Cognitive Impairment and Disability (FINGER) trial^59^ enrolled participants aged 60 to 77 years, and the Advanced Cognitive Training for Independent and Vital Elderly (ACTIVE) trial^60^ enrolled participants older than 65 years. Early enrollment has been proposed as a way forward after disappointing results of recent trials,^61^ but our findings suggest that prevention may need to start 25 or more years before likely age at onset.

### Strengths and Limitations

A strength of this study design is the identification of very early symptoms of AD while avoiding the ambiguity between factors that influence cognitive reserve or ease of diagnosis and those that reflect early symptoms. This design is a feasible approach to evaluate the earliest manifestations of AD without requiring long follow-up. By using cross-validation to choose the threshold age at which the GRS begins to influence cognition data adaptively, our modeling approach provides an estimate of the age at which AD begins to exert influence on each phenotype. In addition, the hypothesis-free approach allows comparison of multiple cognitive indicators to identify the most important early indicators of AD.

This study also has some limitations. First, this study was cross-sectional and interpreted differences in mean cognitive scores between older and younger people and level of AD-GRS. Such cross-sectional age differences represent the combination of biological aging, cohort differences, and any differential selection by age. Measurement error in the cognitive tests also makes it more difficult to detect age-associated changes in test performance. Because there is some evidence of a healthy volunteer effect in the UK Biobank that becomes more pronounced with age,^29,62^ future work examining longitudinal cognitive changes may be informative. Second, we restricted analysis to individuals with European ancestry—the predominant population of previous AD-GRS studies—and our results may not reflect patterns in other populations. Third, the UK Biobank cognitive test battery was not comprehensive and omitted measures that may be sensitive to early cognitive changes in AD, such as tests of episodic memory that capture long-delay recall and recognition,^63,64^ and measures of verbal fluency,^23^ language,^65,66^ and working memory^67^ or other approaches.^68^ With more sensitive measures, we may have detected even earlier changes. Fourth, caution in interpreting test differences is merited because the samples differed in size and composition. With a large sample size, some cognitive measures may have had an earlier detectable age of divergence. Fifth, although useful for improving understanding of the natural history of AD, the mean differences in cognition by AD-GRS are too small to be clinically relevant at younger ages. Sixth, selective survival or selective study participation may have introduced bias. Previous research using the same GRS in the UK Biobank with smoking as a negative control suggested that survival bias is minimal; this AD-GRS did not modify the age association with smoking despite the strong association between smoking and survival.^69^ Although participation in the UK Biobank might be differential for individuals with high AD-GRSs, selection bias would likely attenuate our findings because including more impaired higher AD-GRS cases would improve power to detect early differences by AD-GRS.

## Conclusions

This cross-sectional study adds to the increasing evidence that cognitive outcomes associated with AD genes may begin in early midlife. The results indicate that multiple cognitive changes as early as 40 years of age may be relevant to AD development for some individuals. Research on the biological changes underlying the early cognitive symptoms is needed. Hypothesis-free approaches using genetic profiles have the potential to identify and compare early indicators of AD and other diseases with long preclinical periods.

## Notes

Supplement. **eAppendix 1.** Association Between AD-GRS and Alzheimer Disease Diagnosis in UK Biobank **eAppendix 2.** Variable Descriptions **eAppendix 3.** Cognitive Tests Description **eAppendix 4.** Functional Form and Cross-Validation Details **eAppendix 5.** Sensitivity Analysis AD-GRS With Alternative Functional Forms for Age **eAppendix 6.** Sensitivity Analysis Using Count of APOE ε4 Alleles **eAppendix 7.** Sensitivity Analysis Using Count of APOE ε4 Alleles and an AD-GRS That Excludes APOE **eAppendix 8.** Sensitivity Analysis Excluding APOE From the AD-GRS **eTable 1.** Logistic Regression of AD Diagnosis on *z*-Scored AD-GRS Including APOE **eTable 2.** Logistic Regression of AD Diagnosis on *z*-Scored AD-GRS, Adjusted for Covariates **eTable 3.** SNPs and Their Log Odds Ratio Estimates for the Alzheimer Disease Genetic Risk Score (AD-GRS) from 2013 Lambert, et. al. **eTable 4.** Description of Phenotype Variables **eTable 5.** Association With AD-GRS and Effect Modification of Age-Slope by AD-GRS for Each Cognitive Assessment (*z*-Scored) **eTable 6.** Age of Divergence in Main Analysis **eTable 7.** Association of APOE Count at Age 40 and Effect Modification of Age-Slope by Count of APOE ε4 Alleles for Each Cognitive Assessment (*z*-Scored) **eTable 8.** Age of Divergence in Sensitivity Analysis Excluding APOE From the AD-GRS **eTable 9.** Association of AD-GRS (Excluding APOE) at Age 40 and Effect Modification of Age-Slope by AD-GRS for Each Cognitive Assessment (*z*-Scored) **eTable 10.** Age of Divergence in Sensitivity Analysis Using Count of APOE ε4-Alleles and AD-GRS That Excludes APOE **eTable 11.** Association of AD-GRS (Excluding APOE) at Age 40 and Effect Modification of Age-Slope by AD-GRS for Each Cognitive Assessment (*z*-Scored) **eTable 12.** Age of Divergence in Sensitivity Analysis Excluding APOE From the AD-GRS **eTable 13.** Age of Divergence in Sensitivity Analysis With Linear Age Term and Quadratic Divergence Term **eTable 14.** Age of Divergence in Sensitivity Analysis With Cubic Polynomial Age Term and Fourth-Order Divergence Term **eTable 15.** Comparison of Ages of Divergence Across Different Functional Forms **eFigure 1.** Example Age of Divergence Calculation Cross-Validation Performance Plot for the Number of Attempts on a Numeric Trail-Making Test (in Person) **eFigure 2.** Divergence Plots for All Cognitive Tests – Part 1 **eFigure 3.** Divergence Plots for All Cognitive Tests – Part 2 **eFigure 4.** Mean Cognitive Scores by Age and AD-GRS, Unadjusted for Covariates – Part 1 **eFigure 5.** Mean Cognitive Scores by Age and AD-GRS, Unadjusted for Covariates – Part 2
eReferences
