# Supplementary material for: Association of Genetic Variants Linked to Late-Onset Alzheimer Disease With Cognitive Test Performance by Midlife
Source: JAMA Netw Open. 2022 Apr 4;5(4):e225491. doi: 10.1001/jamanetworkopen.2022.5491 (PMC8980909; doi:10.1001/jamanetworkopen.2022.5491)
Supplement: Supplement. — eAppendix 1. Association Between AD-GRS and Alzheimer Disease Diagnosis in UK Biobank eAppendix 2. Variable Descriptions eAppendix 3. Cognitive Tests Description eAppendix 4. Functional Form and Cross-Validation Details eAppendix 5. Sensitivity Analysis AD-GRS With Alternative Functional Forms for Age eAppendix 6. Sensitivity Analysis Using Count of APOE ε4 Alleles eAppendix 7. Sensitivity Analysis Using Count of APOE ε4 Alleles and an AD-GRS That Excludes APOE eAppendix 8. Sensitivity Analysis Excluding APOE From the AD-GRS eTable 1. Logistic Regression of AD Diagnosis on z-Scored AD-GRS Including APOE eTable 2. Logistic Regression of AD Diagnosis on z-Scored AD-GRS, Adjusted for Covariates eTable 3. SNPs and Their Log Odds Ratio Estimates for the Alzheimer Disease Genetic Risk Score (AD-GRS) from 2013 Lambert, et. al. eTable 4. Description of Phenotype Variables eTable 5. Association With AD-GRS and Effect Modification of Age-Slope by AD-GRS for Each Cognitive Assessment (z-Scored) eTable 6. Age of Divergence in Main Analysis eTable 7. Association of APOE Count at Age 40 and Effect Modification of Age-Slope by Count of APOE ε4 Alleles for Each Cognitive Assessment (z-Scored) eTable 8. Age of Divergence in Sensitivity Analysis Excluding APOE From the AD-GRS eTable 9. Association of AD-GRS (Excluding APOE) at Age 40 and Effect Modification of Age-Slope by AD-GRS for Each Cognitive Assessment (z-Scored) eTable 10. Age of Divergence in Sensitivity Analysis Using Count of APOE ε4-Alleles and AD-GRS That Excludes APOE eTable 11. Association of AD-GRS (Excluding APOE) at Age 40 and Effect Modification of Age-Slope by AD-GRS for Each Cognitive Assessment (z-Scored) eTable 12. Age of Divergence in Sensitivity Analysis Excluding APOE From the AD-GRS eTable 13. Age of Divergence in Sensitivity Analysis With Linear Age Term and Quadratic Divergence Term eTable 14. Age of Divergence in Sensitivity Analysis With Cubic Polynomial Age Term and Fourth-Order Divergence Term eTable [file jamanetwopen-e225491-s001.pdf]

Supplementary Online Content

Zimmerman SC, Brenowitz WD, Calmasini C, et al. Association of genetic variants linked to late-onset Alzheimer disease with cognitive test performance by midlife. *JAMA Netw Open*. 2022;5(4):e225491. doi:10.1001/jamanetworkopen.2022.5491

eAppendix 1. Association Between AD-GRS and Alzheimer's Disease Diagnosis in UK Biobank
eAppendix 2. Variable Descriptions
eAppendix 3. Cognitive Tests Description
eAppendix 4. Functional Form and Cross-Validation Details
eAppendix 5. Sensitivity Analysis AD-GRS With Alternative Functional Forms for Age
eAppendix 6. Sensitivity Analysis Using Count of APOE ε4 Alleles
eAppendix 7. Sensitivity Analysis Using Count of APOE ε4 Alleles and an AD-GRS That Excludes APOE
eAppendix 8. Sensitivity Analysis Excluding APOE From the AD-GRS
eTable 1. Logistic Regression of AD Diagnosis on *z*-Scored AD-GRS Including APOE
eTable 2. Logistic Regression of AD Diagnosis on *z*-Scored AD-GRS, Adjusted for Covariates
eTable 3. SNPs and Their Log Odds Ratio Estimates for the Alzheimer's Disease Genetic Risk Score (AD-GRS) from 2013 Lambert, et. al.[3]
eTable 4. Description of Phenotype Variables
eTable 5. Association With AD-GRS and Effect Modification of Age-Slope by AD-GRS for Each Cognitive Assessment (*z*-Scored)
eTable 6. Age of Divergence in Main Analysis
eTable 7. Association of APOE Count at Age 40 and Effect Modification of Age-Slope by Count of APOE ε4 Alleles for Each Cognitive Assessment (*z*-Scored)
eTable 8. Age of Divergence in Sensitivity Analysis Excluding APOE From the AD-GRS
eTable 9. Association of AD-GRS (Excluding APOE) at Age 40 and Effect Modification of age-Slope by AD-GRS for Each Cognitive Assessment (*z*-Scored)
eTable 10. Age of Divergence in Sensitivity Analysis Using Count of APOE ε4-Alleles and AD-GRS That Excludes APOE
eTable 11. Association of AD-GRS (Excluding APOE) at Age 40 and Effect Modification of Age-Slope by AD-GRS for Each Cognitive Assessment (*z*-Scored)
eTable 12. Age of Divergence in Sensitivity Analysis Excluding APOE From the AD-GRS
eTable 13. Age of Divergence in Sensitivity Analysis With Linear Age Term and Quadratic Divergence Term
eTable 14. Age of Divergence in Sensitivity Analysis With Cubic Polynomial Age Term and Fourth-Order Divergence Term
eTable 15. Comparison of Ages of Divergence Across Different Functional Forms
eFigure 1. Example Age of Divergence Calculation Cross-Validation Performance Plot for the Number of Attempts on a Numeric Trail-Making Test (in Person)
eFigure 2. Divergence Plots for All Cognitive Tests – Part 1
eFigure 3. Divergence Plots for All Cognitive Tests – Part 2
eFigure 4. Mean Cognitive Scores by Age and AD-GRS, Unadjusted for Covariates – Part 1
eFigure 5. Mean Cognitive Scores by Age and AD-GRS, Unadjusted for Covariates – Part 2
eReferences

This supplementary material has been provided by the authors to give readers additional information about their work.

© 2022 Zimmerman SC et al. *JAMA Network Open.*

eAppendix 1: Association between AD-GRS and Alzheimer's Disease diagnosis in UK Biobank

We conducted a multivariate logistic regression to assess the association between AD-GRS and a diagnosis of Alzheimer's disease in UK Biobank.

The ICD codes used to define dementia diagnosis variable were as follows: ICD 10 codes – F00 Dementia in Alzheimer's disease; F00.0 Dementia in Alzheimer's disease with early onset; F00.1 Dementia in Alzheimer's disease with late onset; F00.2 Dementia in Alzheimer's disease, atypical or mixed type; F00.9 Dementia in Alzheimer's disease, unspecified; G30 Alzheimer's disease; G30.0 Alzheimer's disease with early onset; G30.1 Alzheimer's disease with late onset; G30.8 Other Alzheimer's disease; G30.9 Alzheimer's disease unspecified. ICD9 code – 331.0 Alzheimer's disease.

We regressed AD diagnosis variable on z-scored AD-GRS, adjusted for a linear combination of age at cognitive assessment, self-reported sex, genotyping assay (a binary indicator for whether the UK BiLEVE or UK Biobank Axiom array was used), assessment center, and 10 genetic ancestry principal components (PCs) provided by the UK Biobank to account for population stratification in the sample.

The data included 408,833 participants, 632 of whom had a diagnosis of AD. The z-scored AD-GRS showed a highly statistically significant association with the AD-GRS that included APOE ($b=0.848$, $p < 0.001$, eTable 11). The association between the AD-GRS was also statistically significant, but the strength of its relationship with dementia was attenuated ($b=0.250$, $p=0.065$, eTable 8).

© 2022 Zimmerman SC et al. *JAMA Network Open.*

eAppendix 2: Variable descriptions

Codes for other variables
UK Biobank variables (variable code) included: sex (31), genotype measurement batch (22000), assessment center (54), and 10 genetic ancestry PCs (22009_0_1 to 22009_0_10); age at test was derived from: age at baseline (21022), month of birth (52), year of birth (34), date attending assessment center (53), age when attended assessment center (21003), when pairs test completed (20134), when fluid intelligence test completed (20135), when trail making test completed (20136), when symbol digit substitution test completed (20137), when numeric memory test completed (20138).

eAppendix 3: Cognitive tests description

In-person trail making and symbol digit substitution were not conducted until 2016, at the time neuroimaging was added to the study. For all other variables, we used baseline data from the initial assessment visit (2006-2010). Many tests were also administered in an online format. For the online variables where the test was administered in multiple study waves, we used the first available assessment. More information on the tests as fielded by UK Biobank is available the UK Biobank Data Showcase.[1,2]

Fluid intelligence

This logic/reasoning test gave participants two minutes to answer as many questions as possible across 14 (online) or 13 (in person) questions. For comparability, we used the sum of correct answers across the 13 common questions as the phenotype variable. Participants were scored zero for all unattempted questions.

Numeric memory

In this test of short-term memory capacity and attention, the participant was first shown a 2-digit number. After a brief pause, they were asked to recall the digits in the correct order. Each time the participant correctly remembers a sequence, they are shown a new sequence that is one-digit-longer number, until they make an error or 12 digits are reached. The online version had only 1 round, whereas the in-person test repeated this sequence 3 times. For comparability, we used the maximum number of digits remembered correctly in the first round as the phenotype variable.

Pairs matching

Pairs matching is a visual short-term memory and attention task in which participants are shown an arrangement of cards for 5 seconds and are asked to memorize their position. They must then match them from memory. The online version asks participants to match 3, 6, and 8 cards in three separate rounds. The 8 pair round was only presented if they had 0-1 errors in the 6 card round. The in-person version only tested participants on 3 and 6 pairs. Thus we look only at the 3 and 6 pair tests (separately). Participants were allowed to abandon the task. Outcomes for each round were the time to complete the round (there was no time limit), number of correct matches by the end of the round and number of incorrect attempts made in the round.

Symbol digit substitution

This processing speed task presents participants with a key that matches 8 symbols to the numbers 1-8. They are then shown an array of numbers and are asked to draw (“substitute”) the correct symbols for each item in the array by referring to the key. Participants were instructed to perform as many substitutions as they could within a time limit of one minute, working as quickly and accurately as they could. Outcomes are coded as the number of symbol digit matches made correctly, and the time to complete the first 10 substitutions (participants completed different numbers of substitutions, but 77% of participants who attempted the test completed the first 10).

Trail making test, parts A (numeric) and B (alphanumeric)

In part A, participants are asked to draw connections between the numbers 1-25 as quickly as possible. In part B, they are asked to alternate between connecting numbers and letters, i.e. connecting the sequence 1,A,2,B etc. Numeric sequencing is primarily a measure of processing speed, whereas the alphanumeric sequencing requires set-shifting, a subdomain of executive functioning. For each condition, we analyzed the time to complete and the number of errors.

Reaction time

This reaction time task presented participants with two cards at a time. They were asked to respond as quickly as possible if the cards matched. The outcome was mean reaction time to identify matches, excluding training rounds 1-4, in milliseconds.

Prospective memory

For this prospective memory task, participants were asked to perform an action at a future time, “At the end of the games we will show you four coloured shapes and ask you to touch the Blue Square. However, to test your memory, we want you to actually touch the Orange Circle instead.” If participants correctly recalled this instruction and chose the orange circle, they were scored as correct. Any other answer was scored as incorrect.

eAppendix 4: Functional form and cross-validation details

Functional Form

The functional form of the regressions is:

$$Y \sim link(t^2 + t + ADGRS_z \times I(t > t_{threshold}) \times (t - t_{threshold})^3 + \sum_i W_i)$$

Where:

- t is the difference between age at assessment and the reference age (40 years).
- W_i are covariates: age at cognitive assessment, self-reported sex, genotyping assay (a binary indicator for whether a UK BiLEVE or UK Biobank Axiom array was used), assessment center (for assessment-center based measures only), practice effect (for online measures only) and 10 genetic ancestry PCs.
- “link” is the identity transform for continuous variables, and the logit transform for binary variables
- $ADGRS_z$ is the “z-scored” or “standardized” value of each participants genetic risk score obtained from:

  $$ADGRS_z = \frac{ADGRS - \ mean(ADGRS)}{sd(ADGRS)}$$

  Where mean(AD-GRS) and sd(AD-GRS) are the mean and standard deviation AD-GRS scores in the full sample.

Using this functional form, we assume that the mean level of the cognitive measure changes quadratically with age below the threshold, and diverges smoothly (in terms of first derivatives) from this curve as t increases above the threshold.

Cross-validation

For each phenotype we conducted 10-fold cross-validation in which $t_{threshold}$ was varied in 1-year increments over the range of values supported by the age range in the sample for the phenotype in order to choose the value of $t_{threshold}$, the time (relative to the centered reference age) at which the predicted value for individuals with the same covariates, but different AD-GRS, would begin to diverge. The data set was divided randomly into 10 subsets called “folds”. For each possible value of $t_{threshold}$, one cross-validation was conducted using each fold as the test set, and the rest of the sample as the training set, and we measured the mean-squared prediction error (MSPE) for the fold as the mean squared difference in predicted and observed phenotype values. The total error for the model with $t_{threshold}$ was then the sum of these 10 fold-specific MSPEs. We chose $t_{threshold}$ for each phenotype based on which model had the lowest total error, which indicated the best out-of-sample prediction accuracy. For comparability, the same 10 folds were used for each value of $t_{threshold}$.
Additionally, to check the performance of the cross-validation, we visually examined the total error as a function of $t_{threshold}$. We expected smooth U-shaped curves with a single global minimum and no local minima, or monotonic curves indicating that the true minimum lies outside the support of the data. See eFigure 1 for an example.

© 2022 Zimmerman SC et al. *JAMA Network Open.*

eAppendix 5: Sensitivity analysis ADGRS with alternative functional forms for age

Using a more flexible functional form (eEquation 4) for the relationship between age and ADGRS led to the same or earlier estimated ages of divergence for all measures (eTable 14) compared to the quadratic functional form (eTable 6) except the indicator of first or second attempt correct for the in-person version of the prospective memory test (see eTable 15). The more rigid functional form (eEquation 3, eTable 13) did not fit the data as well as the quadratic models (eTable 6), and estimated a mix of earlier and later ages of divergence across measures (eTable 15).

eEquation 3: Linear age term with quadratic divergence term

$$Y \sim link(b_0 + (b_1 t) + b_2\, ADGRS_z \times I(t > t_{threshold}) \times (t - t_{threshold})^2 + \textstyle\sum_i b_i W_i\,) \quad \text{(eEquation 3)}$$

eEquation 4: Cubic age term with 4th-order divergence term

$$Y \sim link(b_0 + (b_1 t + b_2 t^2 + b_3 t^3) + b_4\, ADGRS_z \times I(t > t_{threshold}) \times (t - t_{threshold})^4 + \textstyle\sum_i b_i W_i\,) \quad \text{(eEquation 4)}$$

eAppendix 6: Sensitivity analysis using count of APOE ε4 alleles

In sensitivity analyses using the count of APOE ε4 alleles instead of the AD-GRS, there was evidence that the count of APOE ε4 alleles modified the association of age with 11 measures derived from the pairs matching, symbol digit substitution, numeric memory and trail-making tests (eTable 7), with west-fitting models indicated divergence by age 60 for these measures (eTable 8).

For sensitivity analyses presented in eTables 9 and 10 we used the following functional form:

$$Y \sim link(b_0 + b_1 age + b_2 APOE + b_3 APOE \times age + \textstyle\sum_i b_i W_i\,) \quad \text{(eEquation 1)}$$

© 2022 Zimmerman SC et al. *JAMA Network Open.*

Where cognitive score (Y) is a function of age, count of APOE ε4 alleles (APOE), their interaction, and covariates W_i.

eAppendix 7: Sensitivity analysis using count of APOE ε4-alleles and an AD-GRS that excludes APOE
In sensitivity analyses using both the count of APOE ε4 alleles and the AD-GRS omitting SNPs in the APOE region, there was evidence that the count of APOE ε4 alleles modified the association of age with 2 measures derived from the pairs matching, symbol digit substitution, numeric memory and trail-making tests (eTable 9), but no interactions between age and the AD-GRS were significant. Best-fitting models indicated higher ages of divergence, with 9 measures suggesting divergence by age 63, while 3 measures suggested no divergence at the highest observed age (eTable 10).
For sensitivity analyses presented in eTables 9 and 10 we used the following functional form:

$$Y \sim link(b_0 + b_1 age + b_2 ADGRS_z + b_3 ADGRS_z \times age + b_3 APOE + b_4 APOE \times age + \sum_i b_i W_i) \quad \text{(eEquation 2)}$$

Where cognitive score (Y) is a function of age, z-scored AD-GRS ($ADGRS_z$), count of APOE ε4 alleles (APOE), the interaction of age with AD-GRS, the interaction of age with count of APOE ε4 alleles, and covariates W_i.

Using a more flexible functional form for the relationship between age and AD-GRS led to the same or earlier estimated ages of divergence for all measures except the indicator of first or second attempt correct for the in-person version of the prospective memory test. The more rigid functional form did not fit the data as well as the main divergence models, and estimated a mix of earlier and later ages of divergence across measures (eTables 6, 13, 14 and 15).

eAppendix 8 Sensitivity analysis excluding APOE from the AD-GRS
In sensitivity analyses using an AD-GRS that excluded alleles from the APOE region, the AD-GRS did not significantly modify the association of age with any cognitive measures at the Bonferroni threshold, but the interaction was significant at p<0.05 for 3 cognitive measures (eTable 11). Best-fitting models indicated earliest age of divergence before 50 years for 4 cognitive assessments (eTable 12).

© 2022 Zimmerman SC et al. *JAMA Network Open.*

eTable 1: Logistic regression of AD diagnosis on z-scored AD-GRS including APOE

Term	***Coefficient Estimate (b)***	***SE***	***p-value***
(Intercept)	-17.807	177.387	0.920
ADGRS_z	0.848	0.032	0.000[a]
pc1	0.002	0.027	0.927
pc2	0.003	0.027	0.905
pc3	0.039	0.026	0.143
pc4	0.010	0.020	0.606
pc5	-0.008	0.009	0.402
pc6	0.019	0.025	0.459
pc7	0.021	0.023	0.367
pc8	0.037	0.023	0.104
pc9	-0.006	0.010	0.565
pc10	-0.054	0.020	0.008[a]
base_age_c60	0.228	0.010	0.000[a]
sex	-0.073	0.081	0.369
genoChip_0_0	0.105	0.136	0.440
as.factor(assessment_center)11001	9.203	177.387	0.959
as.factor(assessment_center)11002	7.494	177.389	0.966
as.factor(assessment_center)11003	11.581	177.387	0.948
as.factor(assessment_center)11004	11.522	177.387	0.948
as.factor(assessment_center)11005	11.322	177.387	0.949
as.factor(assessment_center)11006	10.182	177.387	0.954
as.factor(assessment_center)11007	8.577	177.387	0.961
as.factor(assessment_center)11008	10.727	177.387	0.952
as.factor(assessment_center)11009	9.672	177.387	0.957
as.factor(assessment_center)11010	11.033	177.387	0.950

© 2022 Zimmerman SC et al. *JAMA Network Open.*

as.factor(assessment_center)11011	8.811	177.387	0.960
as.factor(assessment_center)11012	8.978	177.388	0.960
as.factor(assessment_center)11013	11.028	177.387	0.950
as.factor(assessment_center)11014	10.729	177.387	0.952
as.factor(assessment_center)11016	8.050	177.387	0.964
as.factor(assessment_center)11017	10.905	177.387	0.951
as.factor(assessment_center)11018	10.218	177.387	0.954
as.factor(assessment_center)11020	7.067	177.389	0.968
as.factor(assessment_center)11021	9.820	177.387	0.956
as.factor(assessment_center)11022	9.341	177.390	0.958
as.factor(assessment_center)11023	10.867	177.390	0.951

[a] statistically significant at significance level 0.05; ADGRS_z: z-scored Alzheimer's disease genetic risk score; pc1-pc10: genetic ancestry principal components; base_age_c60: age at recruitment, centered at 60; sex: female (0) or male (1); genochip_0_0: indicator for UK BiLEVE array (0) or UK Biobank Axiom array (1); assessment_center: UK Biobank assessment center ID.

© 2022 Zimmerman SC et al. *JAMA Network Open.*

eTable 2: Logistic regression of AD diagnosis on z-scored AD-GRS, adjusted for covariates

Term	*Coefficient Estimate (b)*	*SE*	*p-value*
(Intercept)	-17.569	185.858	0.925
ADGRS_z	0.250	0.042	0.000[a]
pc1	-0.002	0.026	0.939
pc2	-0.001	0.027	0.963
pc3	0.039	0.026	0.137
pc4	0.013	0.020	0.502
pc5	-0.010	0.009	0.292
pc6	0.022	0.025	0.381
pc7	0.021	0.023	0.361
pc8	0.035	0.023	0.124
pc9	-0.003	0.010	0.777
pc10	-0.053	0.020	0.008[a]
base_age_c60	0.223	0.010	0.000[a]
sex	-0.039	0.080	0.629
genoChip_0_0	0.150	0.135	0.269
as.factor(assessment_center)11001	9.323	185.859	0.960
as.factor(assessment_center)11002	7.632	185.861	0.967
as.factor(assessment_center)11003	11.647	185.858	0.950
as.factor(assessment_center)11004	11.617	185.858	0.950
as.factor(assessment_center)11005	11.425	185.858	0.951
as.factor(assessment_center)11006	10.287	185.858	0.956
as.factor(assessment_center)11007	8.688	185.858	0.963
as.factor(assessment_center)11008	10.823	185.858	0.954
as.factor(assessment_center)11009	9.789	185.858	0.958

© 2022 Zimmerman SC et al. *JAMA Network Open.*

as.factor(assessment_center)11010	11.124	185.858	0.952
as.factor(assessment_center)11011	8.913	185.858	0.962
as.factor(assessment_center)11012	9.057	185.859	0.961
as.factor(assessment_center)11013	11.114	185.858	0.952
as.factor(assessment_center)11014	10.825	185.858	0.954
as.factor(assessment_center)11016	8.167	185.859	0.965
as.factor(assessment_center)11017	11.032	185.858	0.953
as.factor(assessment_center)11018	10.297	185.858	0.956
as.factor(assessment_center)11020	7.158	185.861	0.969
as.factor(assessment_center)11021	9.902	185.858	0.958
as.factor(assessment_center)11022	9.404	185.861	0.960
as.factor(assessment_center)11023	10.795	185.861	0.954

[a] statistically significant ($p<0.05$); ADGRS_z: z-scored Alzheimer's disease genetic risk score; pc1-pc10: genetic ancestry principal components; base_age_c60: age at recruitment, centered at 60; sex: female (0) or male (1); genochip_0_0: indicator for UK BiLEVE array (0) or UK Biobank Axiom array (1); assessment_center: UK Biobank assessment center ID.

© 2022 Zimmerman SC et al. *JAMA Network Open.*

eTable 3: SNPs and Their Log Odds Ratio Estimates for the Alzheimer's Disease Genetic Risk Score (AD-GRS) from 2013 Lambert, et. Al.[3]

Marker Name	Chromosome	Closest gene	Effect Allele	Effect Estimate
rs6656401	1	*CR1*	A	0.1567
rs35349669	2	*INPP5D*	T	0.0663
rs6733839	2	*BIN1*	T	0.188
rs190982	5	*MEF2C*	G	-0.0799
rs10948363	6	*CD2AP*	G	0.0978
rs11771145	7	*EPHA1*	A	-0.1024
rs1476679	7	*ZCWPW1*	C	-0.0783
rs2718058	7	*NME8*	G	-0.0697
rs28834970	8	*PTK2B*	C	0.0959
rs9331896	8	*CLU*	C	-0.1457
rs10792832	11	*PICALM*	A	-0.1297
rs10838725	11	*CELF1*	C	0.0753
rs11218343	11	*SORL1*	C	-0.2697
rs670139	11	*MS4A4E*	T	0.0803
rs983392	11	*MS4A6A*	G	-0.1084
rs10498633	14	*SLC24A4-RIN3*	T	-0.1044
rs17125944	14	*FERMT2*	C	0.1223
rs8093731	18	*DSG2*	T	-0.6136
rs3865444	19	*CD33*	A	-0.0954
rs4147929	19	*ABCA7*	A	0.1348
rs429358[a]	19	*APOE*	C	1.3503
rs7412 [a]	19	*APOE*	T	-0.3871
rs7274581	20	*CASS4*	C	-0.1390

[a] Excluded in AD-GRS without APOE

© 2022 Zimmerman SC et al. *JAMA Network Open.*

eTable 4: Description of Phenotype Variables

**Cognitive test**	**Analysis variable**	**UK Biobank variables (field ID) used to create analysis variable**
Fluid intelligence	Number of correctly answered items out of F1-F13	In-person: 20016<br>Online: 20191
	Number of attempted items out of F1-F13	In person: 20128<br>Online: 20192 (Not available at this time)
Numeric memory	Maximum digits remembered correctly	In person: 4282<br>Online: 20240
Pairs matching	Number incorrect matches (by round, for rounds 1 and 2)	In person: 399<br>Online: 20132
	Number correct matches (by round, for round 1)	In person: 398<br>Online: 20131
	Time to complete round (by round, for rounds 1 and 2)	In person: 400<br>Online: 20133
Symbol digit substitution	Number of symbol digit matches made correctly	In person: 23324<br>Online: 20159
	Number of symbol digit matches attempted	In person: 23323<br>Online: 20195
	Time to complete the first 10 (among those who got the first 10 correct)	In person: N/A – Time not recorded.<br>Online: 20230 (duration to entering value), 20200 (values wanted), 20229 (values entered)
Trail making	Duration to complete numeric path (trail #1)	In person: 6348<br>Online: 20156
	Duration to complete alphanumeric path (trail #2)	In person: 6350<br>Online: 20157

© 2022 Zimmerman SC et al. *JAMA Network Open.*

	Total errors traversing numeric path (trail #1)	In person: 6349<br>Online: 20247
	Total errors traversing alphanumeric path (trail #2)	In person: 3951<br>Online: 20248
Reaction time	Mean time to correctly identify matches	In person: 20023<br>Online: N/A
Prospective memory	Correct on first attempt	In person: 20018<br>Online: N/A
	Correct on first or second attempt	In person: 20018<br>Online: N/A

© 2022 Zimmerman SC et al. *JAMA Network Open.*

eTable 5: Association with AD-GRS and effect modification of age-slope by AD-GRS for each cognitive assessment (z-scored)

Test	*Variable*	*Location*	*Domain*	*Age slope per decade for person with mean GRS [95% CI] p-value* [a]	*Difference in age slope for person with 1SD higher GRS [95% CI] p-value* [b]	*Percent Difference in mean cognition per decade increase in age for 1 SD higher AD GRS* [c]
26) Trail-making	Number Of Errors In Numeric Trail	In Person	processing speed	-0.122 [-0.145,-0.099] p<2e-16	-0.02 [-0.043,0.003] p=9.0e-02	16.3
12) Pairs Matching	Number Correct (Round 1)	Online	short-term memory/attention	-0.124 [-0.132,-0.115] p<2e-16	-0.014 [-0.023,-0.006] p<2e-16	11.5
28) Trail-making	Number Of Errors In Alphanumeric Trail	In Person	processing speed/executive functioning	-0.213 [-0.236,-0.191] p<2e-16	-0.024 [-0.047,-0.001] p=4.0e-02	11.4
7) Pairs Matching	Number Correct (Round 2)	In Person	short-term memory/attention	-0.093 [-0.097,-0.089] p<2e-16	-0.009 [-0.013,-0.005] p<2e-16	9.4
5) Numeric Memory	Number Correct	Online	short-term memory capacity/attention	-0.18 [-0.189,-0.172] p<2e-16	-0.016 [-0.025,-0.007] p<2e-16	8.8
1) Fluid Intelligence	Number Correct	In Person	logic/reasoning	-0.119 [-0.126,-0.112] p<2e-16	-0.01 [-0.017,-0.003] p=1.0e-02	8.4
4) Numeric Memory	Number Correct	In Person	short-term memory capacity/attention	-0.159 [-0.171,-0.147] p<2e-16	-0.013 [-0.025,-0.001] p=4.0e-02	8.2
6) Pairs Matching	Number Correct (Round 1)	In Person	short-term memory/attention	-0.091 [-0.095,-0.087] p<2e-16	-0.007 [-0.011,-0.003] p<2e-16	7.9

© 2022 Zimmerman SC et al. *JAMA Network Open.*

27) Trail-making	Time to Complete Alphanumeric Trail	In Person	processing speed/executive functioning	-0.473 [-0.495,-0.452] p<2e-16	-0.034 [-0.056,-0.012] p<2e-16	7.2
32) Trail-making	Number Of Errors In Alphanumeric Trail	Online	processing speed/executive functioning	-0.128 [-0.142,-0.114] p<2e-16	-0.008 [-0.022,0.006] p=2.9e-01	6
21) Symbol Digit Substitution	Number Correct	In Person	processing speed	-0.599 [-0.62,-0.579] p<2e-16	-0.035 [-0.056,-0.014] p<2e-16	5.8
20) Symbol Digit Substitution	Number Attempted	In Person	processing speed	-0.621 [-0.642,-0.601] p<2e-16	-0.036 [-0.056,-0.015] p<2e-16	5.7
25) Trail-making	Time to Complete Numeric Trail	In Person	processing speed	-0.416 [-0.438,-0.394] p<2e-16	-0.022 [-0.044,0] p=5.0e-02	5.4
10) Pairs Matching	Time To Complete (Round 1)	In Person	short-term memory/attention	-0.207 [-0.211,-0.203] p<2e-16	-0.011 [-0.015,-0.007] p<2e-16	5.3
3) Fluid Intelligence	Number Correct	Online	logic/reasoning	-0.188 [-0.196,-0.179] p<2e-16	-0.01 [-0.018,-0.002] p=2.0e-02	5.2
14) Pairs Matching	Number Incorrect (Round 2)	Online	short-term memory/attention	-0.217 [-0.24,-0.195] p<2e-16	-0.011 [-0.034,0.012] p=3.4e-01	5.2
13) Pairs Matching	Number Incorrect (Round 1)	Online	short-term memory/attention	-0.188 [-0.197,-0.18] p<2e-16	-0.01 [-0.018,-0.001] p=3.0e-02	5.1
18) Prospective Memory	1st Or 2nd Attempt Correct	In Person	prospective memory	-2.975 [-3.185,-2.765] p<2e-16	-0.141 [-0.349,0.066] p=1.8e-01	4.8

© 2022 Zimmerman SC et al. *JAMA Network Open.*

24) Symbol Digit Substitution	Time To Complete 10 Substitutions	Online	processing speed	-0.521 [-0.53,-0.512] p<2e-16	-0.024 [-0.033,-0.015] p<2e-16	4.5
8) Pairs Matching	Number Incorrect (Round 1)	In Person	short-term memory/attention	-0.144 [-0.148,-0.14] p<2e-16	-0.006 [-0.01,-0.002] p<2e-16	4.4
17) Prospective Memory	1st Attempt Correct	In Person	prospective memory	-1.182 [-1.229,-1.135] p<2e-16	-0.043 [-0.09,0.004] p=7.0e-02	3.6
15) Pairs Matching	Time To Complete (Round 1)	Online	short-term memory/attention	-0.44 [-0.448,-0.432] p<2e-16	-0.015 [-0.023,-0.007] p<2e-16	3.5
16) Pairs Matching	Time To Complete (Round 2)	Online	short-term memory/attention	-0.438 [-0.459,-0.416] p<2e-16	-0.014 [-0.036,0.008] p=2.1e-01	3.3
30) Trail-making	Time to Complete Alphanumeric Trail	Online	processing speed/executive functioning	-0.474 [-0.483,-0.466] p<2e-16	-0.013 [-0.022,-0.005] p<2e-16	2.8
11) Pairs Matching	Time To Complete (Round 2)	In Person	short-term memory/attention	-0.312 [-0.315,-0.308] p<2e-16	-0.008 [-0.012,-0.004] p<2e-16	2.5
22) Symbol Digit Substitution	Number Correct	Online	processing speed	-0.587 [-0.594,-0.579] p<2e-16	-0.014 [-0.022,-0.007] p<2e-16	2.4
23) Symbol Digit Substitution	Number Attempted	Online	processing speed	-0.617 [-0.624,-0.61] p<2e-16	-0.012 [-0.02,-0.005] p<2e-16	2
2) Fluid Intelligence	Number Attempted	In Person	logic/reasoning	-0.175 [-0.182,-0.168] p<2e-16	-0.003 [-0.01,0.004] p=4.1e-01	1.7
9) Pairs Matching	Number Incorrect (Round 2)	In Person	short-term memory/attention	-0.185 [-0.189,-0.181] p<2e-16	-0.002 [-0.006,0.002] p=2.7e-01	1.2

© 2022 Zimmerman SC et al. *JAMA Network Open.*

29) Trail-making	Time to Complete Numeric Trail	Online	processing speed	-0.371 [-0.38,-0.363] p<2e-16	-0.004 [-0.012,0.005] p=4.2e-01	1
19) Reaction Time	Mean Time	In Person	reaction time	-0.373 [-0.377,-0.369] p<2e-16	0.001 [-0.003,0.004] p=7.5e-01	-0.2
31) Trail-making	Number Of Errors In Numeric Trail	Online	processing speed	-0.089 [-0.107,-0.072] p<2e-16	0.002 [-0.015,0.02] p=8.1e-01	-2.4

[a] *Coefficient on age term (b_2) in equation 1*

[b] *Coefficient on $ADGRS_z$×age term (b_3) in equation 1*

[c] *($100*b_3/b_2$) using the coefficients from equation 1*

© 2022 Zimmerman SC et al. *JAMA Network Open.*

eTable 6: Age of divergence in main analysis

Test	*Variable*	*Location*	*Minimum Age Tested*	*Maximum Age Tested*	*Age of Divergence*	*Age of Statistical Significance*
6) Pairs Matching [a]	Number Correct (Round 1)	In Person	41	70	<=41	55
9) Pairs Matching	Number Incorrect (Round 2)	In Person	41	70	<=41	70+
11) Pairs Matching [a]	Time To Complete (Round 2)	In Person	41	70	<=41	70+
2) Fluid Intelligence	Number Attempted	In Person	41	70	42	70+
4) Numeric Memory	Number Correct	In Person	41	70	42	70+
8) Pairs Matching [a]	Number Incorrect (Round 1)	In Person	41	70	45	70+
3) Fluid Intelligence	Number Correct	Online	45.4	69.4	<=45.4	59
5) Numeric Memory [a]	Number Correct	Online	45.4	69.4	<=45.4	60
23) Symbol Digit Substitution [a]	Number Attempted	Online	45.4	69.4	<=45.4	56
22) Symbol Digit Substitution [a]	Number Correct	Online	45.4	69.4	<=45.4	56
30) Trail-making	Time to Complete Alphanumeric Trail	Online	45.4	69.4	<=45.4	56
29) Trail-making	Time to Complete Numeric Trail	Online	45.4	69.4	<=45.4	61
16) Pairs Matching	Time To Complete (Round 2)	Online	45.5	69.5	<=45.5	62
32) Trail-making	Number Of Errors In Alphanumeric Trail	Online	45.5	69.5	<=45.5	62
13) Pairs Matching	Number Incorrect (Round 1)	Online	45.4	69.4	46.4	59
15) Pairs Matching [a]	Time To Complete (Round 1)	Online	45.4	69.4	46.4	56
20) Symbol Digit Substitution [a]	Number Attempted	In Person	47	70	<=47	60
21) Symbol Digit Substitution [a]	Number Correct	In Person	47	70	<=47	62

27) Trail-making	Time to Complete Alphanumeric Trail	In Person	47	70	<=47	61
25) Trail-making	Time to Complete Numeric Trail	In Person	47	70	<=47	61
12) Pairs Matching [a]	Number Correct (Round 1)	Online	45.4	69.4	47.4	58
1) Fluid Intelligence	Number Correct	In Person	41	70	49	70+
24) Symbol Digit Substitution [a]	Time To Complete 10 Substitutions	Online	45.4	69.4	49.4	58
7) Pairs Matching [a]	Number Correct (Round 2)	In Person	41	70	50	60
10) Pairs Matching [a]	Time To Complete (Round 1)	In Person	41	70	56	70+
26) Trail-making	Number Of Errors In Numeric Trail	In Person	47	70	59	66
28) Trail-making	Number Of Errors In Alphanumeric Trail	In Person	47	70	61	68
14) Pairs Matching	Number Incorrect (Round 2)	Online	45.5	69.5	64.5	69
18) Prospective Memory	1st Or 2nd Attempt Correct	In Person	41	70	66	70+
19) Reaction Time	Mean Time	In Person	41	70	68	70+
31) Trail-making	Number Of Errors In Numeric Trail	Online	45.4	69.4	69.4	70+
17) Prospective Memory	1st Attempt Correct	In Person	41	70	70	70+

[a] Significant modification of the effect of age by AD-GRS in linear model

© 2022 Zimmerman SC et al. *JAMA Network Open.*

eTable 7: Association of APOE count at age 40 and effect modification of age-slope by count of APOE ε4 alleles for each cognitive assessment (z-scored)

Test	*Variable*	*Location*	*Domain*	*Age slope per decade for person with mean GRS [95% CI]* [a]	*Difference in age slope for person with an additional copy of APOE e4 (95% CI)* [b]	*Percent Difference in mean cognition per decade increase in age per additional copy of APOE e4* [c]
12) Pairs Matching	Number Correct (Round 1)	Online	short-term memory/attention	-0.115 [-0.125,-0.106] p<2e-16	-0.028 [-0.044,-0.011] p<2e-16	23.8
28) Trail-making	Number Of Errors In Alphanumeric Trail	In Person	processing speed/executive functioning	-0.2 [-0.226,-0.174] p<2e-16	-0.046 [-0.091,-0.001] p=4.0e-02	23
4) Numeric Memory	Number Correct	In Person	short-term memory capacity/attention	-0.151 [-0.165,-0.137] p<2e-16	-0.026 [-0.05,-0.003] p=3.0e-02	17.3
5) Numeric Memory	Number Correct	Online	short-term memory capacity/attention	-0.171 [-0.181,-0.161] p<2e-16	-0.03 [-0.047,-0.013] p<2e-16	17.3
7) Pairs Matching	Number Correct (Round 2)	In Person	short-term memory/attention	-0.088 [-0.093,-0.083] p<2e-16	-0.015 [-0.023,-0.008] p<2e-16	17.3
1) Fluid Intelligence	Number Correct	In Person	logic/reasoning	-0.113 [-0.121,-0.105] p<2e-16	-0.019 [-0.033,-0.006] p=1.0e-02	17.1
26) Trail-making	Number Of Errors In Numeric Trail	In Person	processing speed	-0.116 [-0.143,-0.09] p<2e-16	-0.019 [-0.064,0.026] p=4.0e-01	16.6

© 2022 Zimmerman SC et al. *JAMA Network Open.*

14) Pairs Matching	Number Incorrect (Round 2)	Online	short-term memory/attention	-0.208 [-0.235,-0.182] p<2e-16	-0.03 [-0.075,0.016] p=2.0e-01	14.2
6) Pairs Matching	Number Correct (Round 1)	In Person	short-term memory/attention	-0.087 [-0.092,-0.082] p<2e-16	-0.012 [-0.02,-0.005] p<2e-16	14
3) Fluid Intelligence	Number Correct	Online	logic/reasoning	-0.181 [-0.19,-0.171] p<2e-16	-0.023 [-0.039,-0.007] p<2e-16	12.9
16) Pairs Matching	Time To Complete (Round 2)	Online	short-term memory/attention	-0.422 [-0.447,-0.397] p<2e-16	-0.055 [-0.098,-0.011] p=1.0e-02	12.9
27) Trail-making	Time to Complete Alphanumeric Trail	In Person	processing speed/executive functioning	-0.458 [-0.483,-0.433] p<2e-16	-0.052 [-0.095,-0.009] p=2.0e-02	11.4
10) Pairs Matching	Time To Complete (Round 1)	In Person	short-term memory/attention	-0.2 [-0.205,-0.196] p<2e-16	-0.022 [-0.029,-0.014] p<2e-16	10.8
13) Pairs Matching	Number Incorrect (Round 1)	Online	short-term memory/attention	-0.183 [-0.192,-0.173] p<2e-16	-0.018 [-0.034,-0.002] p=3.0e-02	9.9
8) Pairs Matching	Number Incorrect (Round 1)	In Person	short-term memory/attention	-0.14 [-0.145,-0.136] p<2e-16	-0.013 [-0.021,-0.006] p<2e-16	9.5
24) Symbol Digit Substitution	Time To Complete 10 Substitutions	Online	processing speed	-0.507 [-0.517,-0.496] p<2e-16	-0.048 [-0.065,-0.03] p<2e-16	9.4
18) Prospective Memory	1st Or 2nd Attempt Correct	In Person	prospective memory	-2.898 [-3.144,-2.651] p<2e-16	-0.257 [-0.66,0.146] p=2.1e-01	8.9
20) Symbol Digit Substitution	Number Attempted	In Person	processing speed	-0.606 [-0.63,-0.583] p<2e-16	-0.051 [-0.091,-0.01] p=1.0e-02	8.4

© 2022 Zimmerman SC et al. *JAMA Network Open.*

32) Trail-making	Number Of Errors In Alphanumeric Trail	Online	processing speed/executive functioning	-0.125 [-0.141,-0.108] p<2e-16	-0.01 [-0.038,0.018] p=4.8e-01	8.1
25) Trail-making	Time to Complete Numeric Trail	In Person	processing speed	-0.407 [-0.432,-0.381] p<2e-16	-0.032 [-0.076,0.011] p=1.4e-01	8
21) Symbol Digit Substitution	Number Correct	In Person	processing speed	-0.587 [-0.611,-0.563] p<2e-16	-0.043 [-0.084,-0.003] p=4.0e-02	7.4
30) Trail-making	Time to Complete Alphanumeric Trail	Online	processing speed/executive functioning	-0.465 [-0.474,-0.455] p<2e-16	-0.032 [-0.049,-0.015] p<2e-16	6.9
15) Pairs Matching	Time To Complete (Round 1)	Online	short-term memory/attention	-0.431 [-0.441,-0.422] p<2e-16	-0.028 [-0.044,-0.012] p<2e-16	6.5
2) Fluid Intelligence	Number Attempted	In Person	logic/reasoning	-0.171 [-0.18,-0.163] p<2e-16	-0.01 [-0.024,0.003] p=1.3e-01	6.1
22) Symbol Digit Substitution	Number Correct	Online	processing speed	-0.577 [-0.586,-0.568] p<2e-16	-0.033 [-0.048,-0.018] p<2e-16	5.7
11) Pairs Matching	Time To Complete (Round 2)	In Person	short-term memory/attention	-0.307 [-0.311,-0.302] p<2e-16	-0.016 [-0.024,-0.009] p<2e-16	5.2
17) Prospective Memory	1st Attempt Correct	In Person	prospective memory	-1.164 [-1.219,-1.11] p<2e-16	-0.058 [-0.15,0.033] p=2.1e-01	5
23) Symbol Digit Substitution	Number Attempted	Online	processing speed	-0.608 [-0.617,-0.6] p<2e-16	-0.029 [-0.044,-0.014] p<2e-16	4.8

© 2022 Zimmerman SC et al. *JAMA Network Open.*

9) Pairs Matching	Number Incorrect (Round 2)	In Person	short-term memory/attention	-0.183 [-0.188,-0.179] p<2e-16	-0.006 [-0.013,0.002] p=1.4e-01	3.1
29) Trail-making	Time to Complete Numeric Trail	Online	processing speed	-0.368 [-0.378,-0.358] p<2e-16	-0.01 [-0.027,0.007] p=2.7e-01	2.6
19) Reaction Time	Mean Time	In Person	reaction time	-0.374 [-0.378,-0.37] p<2e-16	0.003 [-0.004,0.01] p=4.0e-01	-0.8
31) Trail-making	Number Of Errors In Numeric Trail	Online	processing speed	-0.093 [-0.114,-0.073] p<2e-16	0.012 [-0.022,0.046] p=5.0e-01	-12.9

[a] *Coefficient on age term (b_2) in eEquation 1*

[b] *Coefficient on APOE×age term (b_3) in eEquation 1*

[c] *($100*b_3/b_2$) using the coefficients from eEquation 1*

[d] *Cognitive measure with significant interaction effect (b_3)*

© 2022 Zimmerman SC et al. *JAMA Network Open.*

eTable 8: Age of divergence in sensitivity analysis excluding APOE from the AD-GRS

Test	*Variable*	*Location*	*Minimum Age Tested*	*Maximum Age Tested*	*Age of Divergence*	*Age of Statistical Significance*
6) Pairs Matching	Number Correct (Round 1)	In Person	41	70	<=41	70+
3) Fluid Intelligence	Number Correct	Online	45.4	69.4	<=45.4	70+
5) Numeric Memory [a]	Number Correct	Online	45.4	69.4	<=45.4	70+
23) Symbol Digit Substitution [a]	Number Attempted	Online	45.4	69.4	<=45.4	70+
22) Symbol Digit Substitution [a]	Number Correct	Online	45.4	69.4	<=45.4	70+
30) Trail-making [a]	Time to Complete Alphanumeric Trail	Online	45.4	69.4	<=45.4	70+
16) Pairs Matching	Time To Complete (Round 2)	Online	45.5	69.5	<=45.5	70+
20) Symbol Digit Substitution	Number Attempted	In Person	47	70	<=47	70+
21) Symbol Digit Substitution	Number Correct	In Person	47	70	<=47	70+
27) Trail-making	Time to Complete Alphanumeric Trail	In Person	47	70	<=47	70+
25) Trail-making	Time to Complete Numeric Trail	In Person	47	70	<=47	70+
12) Pairs Matching [a]	Number Correct (Round 1)	Online	45.4	69.4	49.4	70+
2) Fluid Intelligence	Number Attempted	In Person	41	70	51	70+
28) Trail-making	Number Of Errors In Alphanumeric Trail	In Person	47	70	53	70+
9) Pairs Matching	Number Incorrect (Round 2)	In Person	41	70	54	70+
11) Pairs Matching [a]	Time To Complete (Round 2)	In Person	41	70	54	70+

© 2022 Zimmerman SC et al. *JAMA Network Open.*

1) Fluid Intelligence	Number Correct	In Person	41	70	55	70+
7) Pairs Matching [a]	Number Correct (Round 2)	In Person	41	70	56	70+
24) Symbol Digit Substitution [a]	Time To Complete 10 Substitutions	Online	45.4	69.4	56.4	70+
15) Pairs Matching [a]	Time To Complete (Round 1)	Online	45.4	69.4	57.4	70+
8) Pairs Matching [a]	Number Incorrect (Round 1)	In Person	41	70	58	70+
13) Pairs Matching	Number Incorrect (Round 1)	Online	45.4	69.4	58.4	70+
14) Pairs Matching	Number Incorrect (Round 2)	Online	45.5	69.5	58.5	70+
10) Pairs Matching [a]	Time To Complete (Round 1)	In Person	41	70	60	70+
18) Prospective Memory	1st Or 2nd Attempt Correct	In Person	41	70	61	70+
17) Prospective Memory	1st Attempt Correct	In Person	41	70	62	70+
29) Trail-making	Time to Complete Numeric Trail	Online	45.4	69.4	63.4	70+
19) Reaction Time	Mean Time	In Person	41	70	64	70+
26) Trail-making	Number Of Errors In Numeric Trail	In Person	47	70	67	70+
4) Numeric Memory	Number Correct	In Person	41	70	68	70+
31) Trail-making	Number Of Errors In Numeric Trail	Online	45.4	69.4	69.4	70+
32) Trail-making	Number Of Errors In Alphanumeric Trail	Online	45.5	69.5	69.5	70+

[a] Significant modification of the effect of age by AD-GRS in interaction models

© 2022 Zimmerman SC et al. *JAMA Network Open.*

eTable 9: Association of AD-GRS (excluding APOE) at age 40 and effect modification of age-slope by AD-GRS for each cognitive assessment (z-scored)

Test	*Variable*	*Location*	*Domain*	*Age slope per decade for person with mean GRS [95% CI]* [a]	*Difference in age slope for person with 1SD higher GRS [95% CI]* [b]	*Difference in mean cognition per decade increase in age for 1 SD higher AD GRS* [c]	*Difference in age slope for person with an additional copy of APOE ε4 (95% CI)* [d]	*Percent Difference in mean cognition per decade increase in age per additional copy of APOE ε4* [e]
26) Trail-making	Number Of Errors In Numeric Trail	In Person	processing speed	-0.116 [-0.142,-0.089] p<2e-16	-0.021 [-0.044,0.002] p=7.0e-02	18.1	-0.02 [-0.065,0.025] p=3.9e-01	17.1
32) Trail-making	Number Of Errors In Alphanumeric Trail	Online	processing speed/executive functioning	-0.125 [-0.141,-0.108] p<2e-16	-0.007 [-0.021,0.008] p=3.5e-01	5.4	-0.01 [-0.038,0.018] p=4.8e-01	8
21) Symbol Digit Substitution	Number Correct	In Person	processing speed	-0.586 [-0.61,-0.562] p<2e-16	-0.03 [-0.051,-0.009] p<2e-16	5.1	-0.043 [-0.084,-0.003] p=4.0e-02	7.4
17) Prospective Memory	1st Attempt Correct	In Person	prospective memory	-1.163 [-1.218,-1.109] p<2e-16	-0.055 [-0.103,-0.007] p=3.0e-02	4.7	-0.058 [-0.15,0.033] p=2.1e-01	5
20) Symbol Digit Substitution	Number Attempted	In Person	processing speed	-0.606 [-0.629,-	-0.027 [-0.048,-	4.5	-0.051 [-0.091,-	8.4

© 2022 Zimmerman SC et al. *JAMA Network Open.*

				0.582] p<2e-16	0.007] p=1.0e-02		0.011] p=1.0e-02	
12) Pairs Matching	Number Correct (Round 1)	Online	short-term memory/attention	-0.115 [-0.125,-0.106] p<2e-16	-0.005 [-0.013,0.004] p=2.8e-01	4.1	-0.027 [-0.044,-0.011] p<2e-16	23.9
7) Pairs Matching	Number Correct (Round 2)	In Person	short-term memory/attention	-0.088 [-0.093,-0.083] p<2e-16	-0.004 [-0.008,0.001] p=9.0e-02	4	-0.015 [-0.023,-0.008] p<2e-16	17.3
27) Trail-making	Time to Complete Alphanumeric Trail	In Person	processing speed/executive functioning	-0.458 [-0.483,-0.432] p<2e-16	-0.018 [-0.04,0.004] p=1.2e-01	3.9	-0.052 [-0.095,-0.009] p=2.0e-02	11.4
6) Pairs Matching	Number Correct (Round 1)	In Person	short-term memory/attention	-0.087 [-0.092,-0.082] p<2e-16	-0.003 [-0.007,0.001] p=1.4e-01	3.5	-0.012 [-0.02,-0.005] p<2e-16	14
1) Fluid Intelligence	Number Correct	In Person	logic/reasoning	-0.113 [-0.121,-0.105] p<2e-16	-0.003 [-0.011,0.004] p=3.4e-01	3.1	-0.019 [-0.033,-0.006] p=1.0e-02	17.2
25) Trail-making	Time to Complete Numeric Trail	In Person	processing speed	-0.406 [-0.432,-0.381] p<2e-16	-0.012 [-0.035,0.01] p=2.7e-01	3.1	-0.033 [-0.076,0.011] p=1.4e-01	8.1
5) Numeric Memory	Number Correct	Online	short-term memory capacity/attention	-0.171 [-0.181,-0.161] p<2e-16	-0.004 [-0.013,0.004] p=3.3e-01	2.5	-0.03 [-0.047,-0.013] p<2e-16	17.4

© 2022 Zimmerman SC et al. *JAMA Network Open.*

4) Numeric Memory	Number Correct	In Person	short-term memory capacity/attention	-0.151 [-0.165,-0.137] p<2e-16	-0.003 [-0.016,0.009] p=5.8e-01	2.3	-0.026 [-0.05,-0.003] p=3.0e-02	17.4
14) Pairs Matching	Number Incorrect (Round 2)	Online	short-term memory/attention	-0.208 [-0.235,-0.182] p<2e-16	-0.004 [-0.027,0.019] p=7.2e-01	2	-0.03 [-0.075,0.016] p=2.0e-01	14.3
18) Prospective Memory	1st Or 2nd Attempt Correct	In Person	prospective memory	-2.896 [-3.143,-2.649] p<2e-16	-0.053 [-0.268,0.162] p=6.3e-01	1.8	-0.256 [-0.659,0.147] p=2.1e-01	8.8
28) Trail-making	Number Of Errors In Alphanumeric Trail	In Person	processing speed/executive functioning	-0.2 [-0.226,-0.174] p<2e-16	-0.003 [-0.026,0.02] p=8.0e-01	1.4	-0.046 [-0.091,-0.001] p=4.0e-02	22.9
10) Pairs Matching	Time To Complete (Round 1)	In Person	short-term memory/attention	-0.2 [-0.205,-0.195] p<2e-16	-0.002 [-0.006,0.002] p=2.9e-01	1.1	-0.022 [-0.029,-0.014] p<2e-16	10.8
24) Symbol Digit Substitution	Time To Complete 10 Substitutions	Online	processing speed	-0.507 [-0.517,-0.496] p<2e-16	-0.004 [-0.013,0.005] p=3.5e-01	0.9	-0.048 [-0.065,-0.03] p<2e-16	9.4
19) Reaction Time	Mean Time	In Person	reaction time	-0.374 [-0.378,-0.37] p<2e-16	-0.003 [-0.006,0.001] p=2.0e-01	0.7	0.003 [-0.004,0.01] p=4.0e-01	-0.8
15) Pairs Matching	Time To Complete (Round 1)	Online	short-term memory/attention	-0.431 [-0.441,-	-0.003 [-0.011,0.006] p=5.3e-01	0.6	-0.028 [-0.044,-	6.5

© 2022 Zimmerman SC et al. *JAMA Network Open.*

				0.422] p<2e-16			0.012] p<2e-16	
8) Pairs Matching	Number Incorrect (Round 1)	In Person	short-term memory/attention	-0.14 [-0.145,-0.136] p<2e-16	-0.001 [-0.005,0.003] p=7.4e-01	0.5	-0.013 [-0.021,-0.006] p<2e-16	9.5
11) Pairs Matching	Time To Complete (Round 2)	In Person	short-term memory/attention	-0.307 [-0.311,-0.302] p<2e-16	-0.001 [-0.005,0.003] p=5.8e-01	0.4	-0.016 [-0.024,-0.009] p<2e-16	5.2
9) Pairs Matching	Number Incorrect (Round 2)	In Person	short-term memory/attention	-0.183 [-0.188,-0.179] p<2e-16	0 [-0.004,0.004] p=1.0e+00	0	-0.006 [-0.013,0.002] p=1.4e-01	3.1
22) Symbol Digit Substitution	Number Correct	Online	processing speed	-0.577 [-0.586,-0.568] p<2e-16	0.001 [-0.007,0.009] p=8.0e-01	-0.2	-0.033 [-0.048,-0.018] p<2e-16	5.7
23) Symbol Digit Substitution	Number Attempted	Online	processing speed	-0.608 [-0.617,-0.6] p<2e-16	0.001 [-0.007,0.009] p=8.1e-01	-0.2	-0.029 [-0.044,-0.014] p<2e-16	4.8
29) Trail-making	Time to Complete Numeric Trail	Online	processing speed	-0.368 [-0.378,-0.358] p<2e-16	0.003 [-0.006,0.011] p=5.8e-01	-0.7	-0.01 [-0.027,0.007] p=2.7e-01	2.6
3) Fluid Intelligence	Number Correct	Online	logic/reasoning	-0.181 [-0.19,-0.171] p<2e-16	0.002 [-0.007,0.01] p=6.7e-01	-1	-0.023 [-0.039,-0.007] p<2e-16	12.8

© 2022 Zimmerman SC et al. *JAMA Network Open.*

30) Trail-making	Time to Complete Alphanumeric Trail	Online	processing speed/executive functioning	-0.465 [-0.474,-0.455] p<2e-16	0.005 [-0.003,0.014] p=2.3e-01	-1.1	-0.032 [-0.049,-0.015] p<2e-16	6.9
13) Pairs Matching	Number Incorrect (Round 1)	Online	short-term memory/attention	-0.183 [-0.192,-0.173] p<2e-16	0.002 [-0.006,0.011] p=6.0e-01	-1.2	-0.018 [-0.034,-0.002] p=3.0e-02	9.9
31) Trail-making	Number Of Errors In Numeric Trail	Online	processing speed	-0.093 [-0.114,-0.073] p<2e-16	0.001 [-0.017,0.019] p=8.9e-01	-1.4	0.012 [-0.022,0.046] p=5.0e-01	-12.9
2) Fluid Intelligence	Number Attempted	In Person	logic/reasoning	-0.172 [-0.18,-0.163] p<2e-16	0.005 [-0.002,0.012] p=1.7e-01	-2.9	-0.01 [-0.024,0.003] p=1.4e-01	6
16) Pairs Matching	Time To Complete (Round 2)	Online	short-term memory/attention	-0.422 [-0.447,-0.397] p<2e-16	0.012 [-0.01,0.034] p=2.7e-01	-2.9	-0.055 [-0.098,-0.011] p=1.0e-02	13

[a] *Coefficient on age term (b_2) in eEquation 2*
[b] *Coefficient on ADGRS$_z$×age term (b_3) in eEquation 2*
[c] *(100*b_3/b_e) using the coefficients from eEquation 2*
[d] *Coefficient on APOE×age term (b_3) in eEquation 2*
[e] *(100*b_5/b_4) using the coefficients from eEquation 2*

© 2022 Zimmerman SC et al. *JAMA Network Open.*

eTable 10: Age of divergence in sensitivity analysis using count of APOE ε4-alleles and an AD-GRS that excludes APOE

Test	*Variable*	*Location*	*Minimum Age Tested*	*Maximum Age Tested*	*Age of Divergence*	*Age of Statistical Significance*
3) Fluid Intelligence	Number Correct	Online	45.4	69.4	<=45.4	63
13) Pairs Matching	Number Incorrect (Round 1)	Online	45.4	69.4	<=45.4	70+
25) Trail-making	Time to Complete Numeric Trail	In Person	47	70	<=47	70+
26) Trail-making	Number Of Errors In Numeric Trail	In Person	47	70	48	65
23) Symbol Digit Substitution [a]	Number Attempted	Online	45.4	69.4	50.4	67
12) Pairs Matching [a]	Number Correct (Round 1)	Online	45.4	69.4	52.4	65
22) Symbol Digit Substitution [a]	Number Correct	Online	45.4	69.4	53.4	66
15) Pairs Matching [a]	Time To Complete (Round 1)	Online	45.4	69.4	54.4	64
32) Trail-making	Number Of Errors In Alphanumeric Trail	Online	45.5	69.5	55.5	67
10) Pairs Matching [a]	Time To Complete (Round 1)	In Person	41	70	57	70+
11) Pairs Matching [a]	Time To Complete (Round 2)	In Person	41	70	57	70+
6) Pairs Matching [a]	Number Correct (Round 1)	In Person	41	70	61	70+
7) Pairs Matching [a]	Number Correct (Round 2)	In Person	41	70	61	70+
24) Symbol Digit Substitution [a]	Time To Complete 10 Substitutions	Online	45.4	69.4	62.4	68
9) Pairs Matching	Number Incorrect (Round 2)	In Person	41	70	64	70+
31) Trail-making	Number Of Errors In Numeric Trail	Online	45.4	69.4	64.4	70+

14) Pairs Matching	Number Incorrect (Round 2)	Online	45.5	69.5	64.5	70+
16) Pairs Matching	Time To Complete (Round 2)	Online	45.5	69.5	64.5	70+
27) Trail-making	Time to Complete Alphanumeric Trail	In Person	47	70	66	70+
28) Trail-making	Number Of Errors In Alphanumeric Trail	In Person	47	70	67	70+
2) Fluid Intelligence	Number Attempted	In Person	41	70	68	70+
4) Numeric Memory	Number Correct	In Person	41	70	68	70+
19) Reaction Time	Mean Time	In Person	41	70	69	70+
5) Numeric Memory [a]	Number Correct	Online	45.4	69.4	69.4	70+
30) Trail-making [a]	Time to Complete Alphanumeric Trail	Online	45.4	69.4	69.4	70+
29) Trail-making	Time to Complete Numeric Trail	Online	45.4	69.4	69.4	70+
1) Fluid Intelligence	Number Correct	In Person	41	70	70	70+
8) Pairs Matching [a]	Number Incorrect (Round 1)	In Person	41	70	70	70+
17) Prospective Memory	1st Attempt Correct	In Person	41	70	70	70+
18) Prospective Memory	1st Or 2nd Attempt Correct	In Person	41	70	70	70+
20) Symbol Digit Substitution	Number Attempted	In Person	47	70	70	70+
21) Symbol Digit Substitution	Number Correct	In Person	47	70	70	70+

© 2022 Zimmerman SC et al. *JAMA Network Open.*

eTable 11: Association of AD-GRS (excluding APOE) at age 40 and effect modification of age-slope by AD-GRS for each cognitive assessment (z-scored)

Test	*Variable*	*Location*	*Domain*	*Age slope per decade for person with mean GRS [95% CI]* [a]	*Difference in age slope for person with 1SD higher GRS [95% CI]* [b]	*Percent Difference in mean cognition per decade increase in age for 1 SD higher AD GRS* [c]
26) Trail-making	Number Of Errors In Numeric Trail	In Person	processing speed	-0.121 [-0.143,-0.098] p<2e-16	-0.021 [-0.044,0.002] p=8.0e-02	17.1
32) Trail-making	Number Of Errors In Alphanumeric Trail	Online	processing speed/executive functioning	-0.128 [-0.142,-0.114] p<2e-16	-0.007 [-0.021,0.008] p=3.5e-01	5.4
21) Symbol Digit Substitution	Number Correct	In Person	processing speed	-0.598 [-0.618,-0.577] p<2e-16	-0.03 [-0.05,-0.009] p=1.0e-02	4.9
17) Prospective Memory	1st Attempt Correct	In Person	prospective memory	-1.181 [-1.228,-1.134] p<2e-16	-0.055 [-0.103,-0.007] p=2.0e-02	4.6
20) Symbol Digit Substitution	Number Attempted	In Person	processing speed	-0.62 [-0.64,-0.599] p<2e-16	-0.027 [-0.047,-0.006] p=1.0e-02	4.3
7) Pairs Matching	Number Correct (Round 2)	In Person	short-term memory/attention	-0.093 [-0.097,-0.089] p<2e-16	-0.004 [-0.008,0] p=9.0e-02	3.8
12) Pairs Matching	Number Correct (Round 1)	Online	short-term memory/attention	-0.123 [-0.131,-0.115] p<2e-16	-0.005 [-0.013,0.004] p=2.8e-01	3.8

© 2022 Zimmerman SC et al. *JAMA Network Open.*

27) Trail-making	Time to Complete Alphanumeric Trail	In Person	processing speed/executive functioning	-0.472 [-0.494,-0.45] p<2e-16	-0.017 [-0.039,0.005] p=1.3e-01	3.6
6) Pairs Matching	Number Correct (Round 1)	In Person	short-term memory/attention	-0.091 [-0.095,-0.087] p<2e-16	-0.003 [-0.007,0.001] p=1.4e-01	3.3
1) Fluid Intelligence	Number Correct	In Person	logic/reasoning	-0.119 [-0.126,-0.112] p<2e-16	-0.003 [-0.011,0.004] p=3.4e-01	2.9
25) Trail-making	Time to Complete Numeric Trail	In Person	processing speed	-0.415 [-0.437,-0.393] p<2e-16	-0.012 [-0.034,0.01] p=2.9e-01	2.9
5) Numeric Memory	Number Correct	Online	short-term memory capacity/attention	-0.18 [-0.188,-0.171] p<2e-16	-0.004 [-0.013,0.004] p=3.3e-01	2.4
4) Numeric Memory	Number Correct	In Person	short-term memory capacity/attention	-0.159 [-0.171,-0.147] p<2e-16	-0.003 [-0.016,0.009] p=5.9e-01	2.2
14) Pairs Matching	Number Incorrect (Round 2)	Online	short-term memory/attention	-0.216 [-0.239,-0.193] p<2e-16	-0.004 [-0.027,0.019] p=7.3e-01	1.9
18) Prospective Memory	1st Or 2nd Attempt Correct	In Person	prospective memory	-2.978 [-3.188,-2.768] p<2e-16	-0.054 [-0.27,0.161] p=6.2e-01	1.8
28) Trail-making	Number Of Errors In Alphanumeric Trail	In Person	processing speed/executive functioning	-0.213 [-0.236,-0.19] p<2e-16	-0.003 [-0.025,0.02] p=8.2e-01	1.2
10) Pairs Matching	Time To Complete (Round 1)	In Person	short-term memory/attention	-0.207 [-0.211,-0.203] p<2e-16	-0.002 [-0.006,0.002] p=2.9e-01	1.1

© 2022 Zimmerman SC et al. *JAMA Network Open.*

24) Symbol Digit Substitution	Time To Complete 10 Substitutions	Online	processing speed	-0.52 [-0.529,-0.511] p<2e-16	-0.004 [-0.013,0.005] p=3.6e-01	0.8
19) Reaction Time	Mean Time	In Person	reaction time	-0.373 [-0.377,-0.369] p<2e-16	-0.003 [-0.006,0.001] p=2.0e-01	0.7
15) Pairs Matching	Time To Complete (Round 1)	Online	short-term memory/attention	-0.439 [-0.447,-0.431] p<2e-16	-0.003 [-0.011,0.006] p=5.3e-01	0.6
8) Pairs Matching	Number Incorrect (Round 1)	In Person	short-term memory/attention	-0.144 [-0.148,-0.14] p<2e-16	-0.001 [-0.005,0.003] p=7.4e-01	0.5
11) Pairs Matching	Time To Complete (Round 2)	In Person	short-term memory/attention	-0.312 [-0.315,-0.308] p<2e-16	-0.001 [-0.005,0.003] p=5.7e-01	0.4
9) Pairs Matching	Number Incorrect (Round 2)	In Person	short-term memory/attention	-0.185 [-0.189,-0.181] p<2e-16	0 [-0.004,0.004] p=9.9e-01	0
22) Symbol Digit Substitution	Number Correct	Online	processing speed	-0.586 [-0.593,-0.578] p<2e-16	0.001 [-0.007,0.009] p=8.0e-01	-0.2
23) Symbol Digit Substitution	Number Attempted	Online	processing speed	-0.616 [-0.624,-0.609] p<2e-16	0.001 [-0.007,0.009] p=8.1e-01	-0.2
29) Trail-making	Time to Complete Numeric Trail	Online	processing speed	-0.371 [-0.38,-0.362] p<2e-16	0.003 [-0.006,0.011] p=5.7e-01	-0.7
3) Fluid Intelligence	Number Correct	Online	logic/reasoning	-0.187 [-0.195,-0.179] p<2e-16	0.002 [-0.007,0.01] p=6.8e-01	-0.9

© 2022 Zimmerman SC et al. *JAMA Network Open.*

30) Trail-making	Time to Complete Alphanumeric Trail	Online	processing speed/executive functioning	-0.474 [-0.482,-0.465] p<2e-16	0.005 [-0.003,0.014] p=2.3e-01	-1.1
13) Pairs Matching	Number Incorrect (Round 1)	Online	short-term memory/attention	-0.188 [-0.196,-0.18] p<2e-16	0.002 [-0.006,0.011] p=6.0e-01	-1.2
31) Trail-making	Number Of Errors In Numeric Trail	Online	processing speed	-0.09 [-0.107,-0.072] p<2e-16	0.001 [-0.017,0.019] p=8.9e-01	-1.5
2) Fluid Intelligence	Number Attempted	In Person	logic/reasoning	-0.175 [-0.182,-0.168] p<2e-16	0.005 [-0.002,0.012] p=1.7e-01	-2.9
16) Pairs Matching	Time To Complete (Round 2)	Online	short-term memory/attention	-0.437 [-0.458,-0.415] p<2e-16	0.013 [-0.01,0.035] p=2.7e-01	-2.9

© 2022 Zimmerman SC et al. *JAMA Network Open.*

[a] *Coefficient on age term (b_2) in equation 1*
[b] *Coefficient on $ADGRS_z$×age term (b_3) in equation 1*
[c] *($100*b_2/b_3$) using the coefficients from equation 1*

eTable 12: Age of divergence in sensitivity analysis excluding APOE from the AD-GRS

Test	***Variable***	***Location***	***Minimum Age Tested***	***Maximum Age Tested***	***Age of Divergence***	***Age of Statistical Significance***
3) Fluid Intelligence	Number Correct	Online	45.4	69.4	<=45.4	63
13) Pairs Matching	Number Incorrect (Round 1)	Online	45.4	69.4	<=45.4	70+
25) Trail-making	Time to Complete Numeric Trail	In Person	47	70	<=47	70+
26) Trail-making	Number Of Errors In Numeric Trail	In Person	47	70	48	65
23) Symbol Digit Substitution	Number Attempted	Online	45.4	69.4	50.4	67
12) Pairs Matching	Number Correct (Round 1)	Online	45.4	69.4	52.4	65
22) Symbol Digit Substitution	Number Correct	Online	45.4	69.4	53.4	66
15) Pairs Matching	Time To Complete (Round 1)	Online	45.4	69.4	54.4	64
32) Trail-making	Number Of Errors In Alphanumeric Trail	Online	45.5	69.5	55.5	67
10) Pairs Matching	Time To Complete (Round 1)	In Person	41	70	57	70+
11) Pairs Matching	Time To Complete (Round 2)	In Person	41	70	57	70+
6) Pairs Matching	Number Correct (Round 1)	In Person	41	70	61	70+
7) Pairs Matching	Number Correct (Round 2)	In Person	41	70	61	70+
24) Symbol Digit Substitution	Time To Complete 10 Substitutions	Online	45.4	69.4	62.4	68
16) Pairs Matching	Time To Complete (Round 2)	Online	45.5	69.5	63.5	70+
9) Pairs Matching	Number Incorrect (Round 2)	In Person	41	70	64	70+
31) Trail-making	Number Of Errors In Numeric Trail	Online	45.4	69.4	64.4	70+
14) Pairs Matching	Number Incorrect (Round 2)	Online	45.5	69.5	64.5	70+
28) Trail-making	Number Of Errors In Alphanumeric Trail	In Person	47	70	67	70+
27) Trail-making	Time to Complete Alphanumeric Trail	In Person	47	70	67	70+

2) Fluid Intelligence	Number Attempted	In Person	41	70	68	70+
4) Numeric Memory	Number Correct	In Person	41	70	68	70+
19) Reaction Time	Mean Time	In Person	41	70	69	70+
5) Numeric Memory	Number Correct	Online	45.4	69.4	69.4	70+
30) Trail-making	Time to Complete Alphanumeric Trail	Online	45.4	69.4	69.4	70+
29) Trail-making	Time to Complete Numeric Trail	Online	45.4	69.4	69.4	70+
1) Fluid Intelligence	Number Correct	In Person	41	70	70	70+
8) Pairs Matching	Number Incorrect (Round 1)	In Person	41	70	70	70+
17) Prospective Memory	1st Attempt Correct	In Person	41	70	70	70+
18) Prospective Memory	1st Or 2nd Attempt Correct	In Person	41	70	70	70+
20) Symbol Digit Substitution	Number Attempted	In Person	47	70	70	70+
21) Symbol Digit Substitution	Number Correct	In Person	47	70	70	70+

© 2022 Zimmerman SC et al. *JAMA Network Open.*

eTable 13: Age of divergence in sensitivity analysis with linear age term and quadratic divergence term

Test	*Variable*	*Location*	*Minimum Age Tested*	*Maximum Age Tested*	*Age of Divergence*	*Age of Statistical Significance*
8) Pairs Matching	Number Incorrect (Round 1)	In Person	41	70	<=41	70+
9) Pairs Matching	Number Incorrect (Round 2)	In Person	41	70	<=41	70+
11) Pairs Matching	Time To Complete (Round 2)	In Person	41	70	<=41	70+
19) Reaction Time	Mean Time	In Person	41	70	<=41	70+
10) Pairs Matching	Time To Complete (Round 1)	In Person	41	70	43	70+
4) Numeric Memory	Number Correct	In Person	41	70	45	70+
13) Pairs Matching	Number Incorrect (Round 1)	Online	45.4	69.4	<=45.4	54
15) Pairs Matching	Time To Complete (Round 1)	Online	45.4	69.4	<=45.4	52
23) Symbol Digit Substitution	Number Attempted	Online	45.4	69.4	<=45.4	52
22) Symbol Digit Substitution	Number Correct	Online	45.4	69.4	<=45.4	52
24) Symbol Digit Substitution	Time To Complete 10 Substitutions	Online	45.4	69.4	<=45.4	51
30) Trail-making	Time to Complete Alphanumeric Trail	Online	45.4	69.4	<=45.4	52
29) Trail-making	Time to Complete Numeric Trail	Online	45.4	69.4	<=45.4	57
14) Pairs Matching	Number Incorrect (Round 2)	Online	45.5	69.5	<=45.5	57
16) Pairs Matching	Time To Complete (Round 2)	Online	45.5	69.5	<=45.5	57
32) Trail-making	Number Of Errors In Alphanumeric Trail	Online	45.5	69.5	<=45.5	58
6) Pairs Matching	Number Correct (Round 1)	In Person	41	70	46	54
7) Pairs Matching	Number Correct (Round 2)	In Person	41	70	46	54
20) Symbol Digit Substitution	Number Attempted	In Person	47	70	<=47	56
21) Symbol Digit Substitution	Number Correct	In Person	47	70	<=47	58

© 2022 Zimmerman SC et al. *JAMA Network Open.*

27) Trail-making	Time to Complete Alphanumeric Trail	In Person	47	70	<=47	56
2) Fluid Intelligence	Number Attempted	In Person	41	70	48	70+
28) Trail-making	Number Of Errors In Alphanumeric Trail	In Person	47	70	49	58
25) Trail-making	Time to Complete Numeric Trail	In Person	47	70	49	58
1) Fluid Intelligence	Number Correct	In Person	41	70	51	70+
5) Numeric Memory	Number Correct	Online	45.4	69.4	51.4	60
12) Pairs Matching	Number Correct (Round 1)	Online	45.4	69.4	51.4	57
3) Fluid Intelligence	Number Correct	Online	45.4	69.4	53.4	60
31) Trail-making	Number Of Errors In Numeric Trail	Online	45.4	69.4	53.4	70+
26) Trail-making	Number Of Errors In Numeric Trail	In Person	47	70	55	61
18) Prospective Memory	1st Or 2nd Attempt Correct	In Person	41	70	67	70+
17) Prospective Memory	1st Attempt Correct	In Person	41	70	70	70+

© 2022 Zimmerman SC et al. *JAMA Network Open.*

eTable 14: Age of divergence in sensitivity analysis with cubic polynomial age term and fourth-order divergence term

Test	***Variable***	***Location***	***Minimum Age Tested***	***Maximum Age Tested***	***Age of Divergence***	***Age of Statistical Significance***
2) Fluid Intelligence	Number Attempted	In Person	41	70	<=41	70+
4) Numeric Memory	Number Correct	In Person	41	70	<=41	70+
6) Pairs Matching	Number Correct (Round 1)	In Person	41	70	<=41	58
7) Pairs Matching	Number Correct (Round 2)	In Person	41	70	<=41	57
8) Pairs Matching	Number Incorrect (Round 1)	In Person	41	70	<=41	70+
9) Pairs Matching	Number Incorrect (Round 2)	In Person	41	70	<=41	70+
10) Pairs Matching	Time To Complete (Round 1)	In Person	41	70	<=41	70+
11) Pairs Matching	Time To Complete (Round 2)	In Person	41	70	<=41	70+
19) Reaction Time	Mean Time	In Person	41	70	<=41	70+
3) Fluid Intelligence	Number Correct	Online	45.4	69.4	<=45.4	61
5) Numeric Memory	Number Correct	Online	45.4	69.4	<=45.4	62
12) Pairs Matching	Number Correct (Round 1)	Online	45.4	69.4	<=45.4	60
13) Pairs Matching	Number Incorrect (Round 1)	Online	45.4	69.4	<=45.4	61
15) Pairs Matching	Time To Complete (Round 1)	Online	45.4	69.4	<=45.4	58
23) Symbol Digit Substitution	Number Attempted	Online	45.4	69.4	<=45.4	59
22) Symbol Digit Substitution	Number Correct	Online	45.4	69.4	<=45.4	59
24) Symbol Digit Substitution	Time To Complete 10 Substitutions	Online	45.4	69.4	<=45.4	58
31) Trail-making	Number Of Errors In Numeric Trail	Online	45.4	69.4	<=45.4	70+
30) Trail-making	Time to Complete Alphanumeric Trail	Online	45.4	69.4	<=45.4	59

© 2022 Zimmerman SC et al. *JAMA Network Open.*

29) Trail-making	Time to Complete Numeric Trail	Online	45.4	69.4	<=45.4	62
14) Pairs Matching	Number Incorrect (Round 2)	Online	45.5	69.5	<=45.5	62
16) Pairs Matching	Time To Complete (Round 2)	Online	45.5	69.5	<=45.5	63
32) Trail-making	Number Of Errors In Alphanumeric Trail	Online	45.5	69.5	<=45.5	64
1) Fluid Intelligence	Number Correct	In Person	41	70	47	70+
20) Symbol Digit Substitution	Number Attempted	In Person	47	70	<=47	63
21) Symbol Digit Substitution	Number Correct	In Person	47	70	<=47	65
28) Trail-making	Number Of Errors In Alphanumeric Trail	In Person	47	70	<=47	63
26) Trail-making	Number Of Errors In Numeric Trail	In Person	47	70	<=47	62
27) Trail-making	Time to Complete Alphanumeric Trail	In Person	47	70	<=47	63
25) Trail-making	Time to Complete Numeric Trail	In Person	47	70	<=47	63
17) Prospective Memory	1st Attempt Correct	In Person	41	70	70	70+
18) Prospective Memory	1st Or 2nd Attempt Correct	In Person	41	70	70	70+

© 2022 Zimmerman SC et al. *JAMA Network Open.*

eTable 15: Comparison of ages of divergence across different functional forms

Test	*Linear*	*Quadratic*	*Cubic*
6) Pairs Matching	46	<=41	<=41
9) Pairs Matching	<=41	<=41	<=41
11) Pairs Matching	<=41	<=41	<=41
2) Fluid Intelligence	48	42	<=41
4) Numeric Memory	45	42	<=41
8) Pairs Matching	<=41	45	<=41
3) Fluid Intelligence	53.4	<=45.4	<=45.4
5) Numeric Memory	51.4	<=45.4	<=45.4
23) Symbol Digit Substitution	<=45.4	<=45.4	<=45.4
22) Symbol Digit Substitution	<=45.4	<=45.4	<=45.4
30) Trail-making	<=45.4	<=45.4	<=45.4
29) Trail-making	<=45.4	<=45.4	<=45.4
16) Pairs Matching	<=45.5	<=45.5	<=45.5
32) Trail-making	<=45.5	<=45.5	<=45.5
13) Pairs Matching	<=45.4	46.4	<=45.4
15) Pairs Matching	<=45.4	46.4	<=45.4
20) Symbol Digit Substitution	<=47	<=47	<=47
21) Symbol Digit Substitution	<=47	<=47	<=47
27) Trail-making	<=47	<=47	<=47
25) Trail-making	49	<=47	<=47
12) Pairs Matching	51.4	47.4	<=45.4
1) Fluid Intelligence	51	49	47
24) Symbol Digit Substitution	<=45.4	49.4	<=45.4

© 2022 Zimmerman SC et al. *JAMA Network Open.*

7) Pairs Matching	46	50	<=41
10) Pairs Matching	43	56	<=41
26) Trail-making	55	59	<=47
28) Trail-making	49	61	<=47
14) Pairs Matching	<=45.5	64.5	<=45.5
18) Prospective Memory	67	66	70
19) Reaction Time	<=41	68	<=41
31) Trail-making	53.4	69.4	<=45.4
17) Prospective Memory	70	70	70

© 2022 Zimmerman SC et al. *JAMA Network Open.*

eFigure 1: Example age of divergence calculation cross-validation performance plot for the number of attempts on a numeric trail-making test (in person).

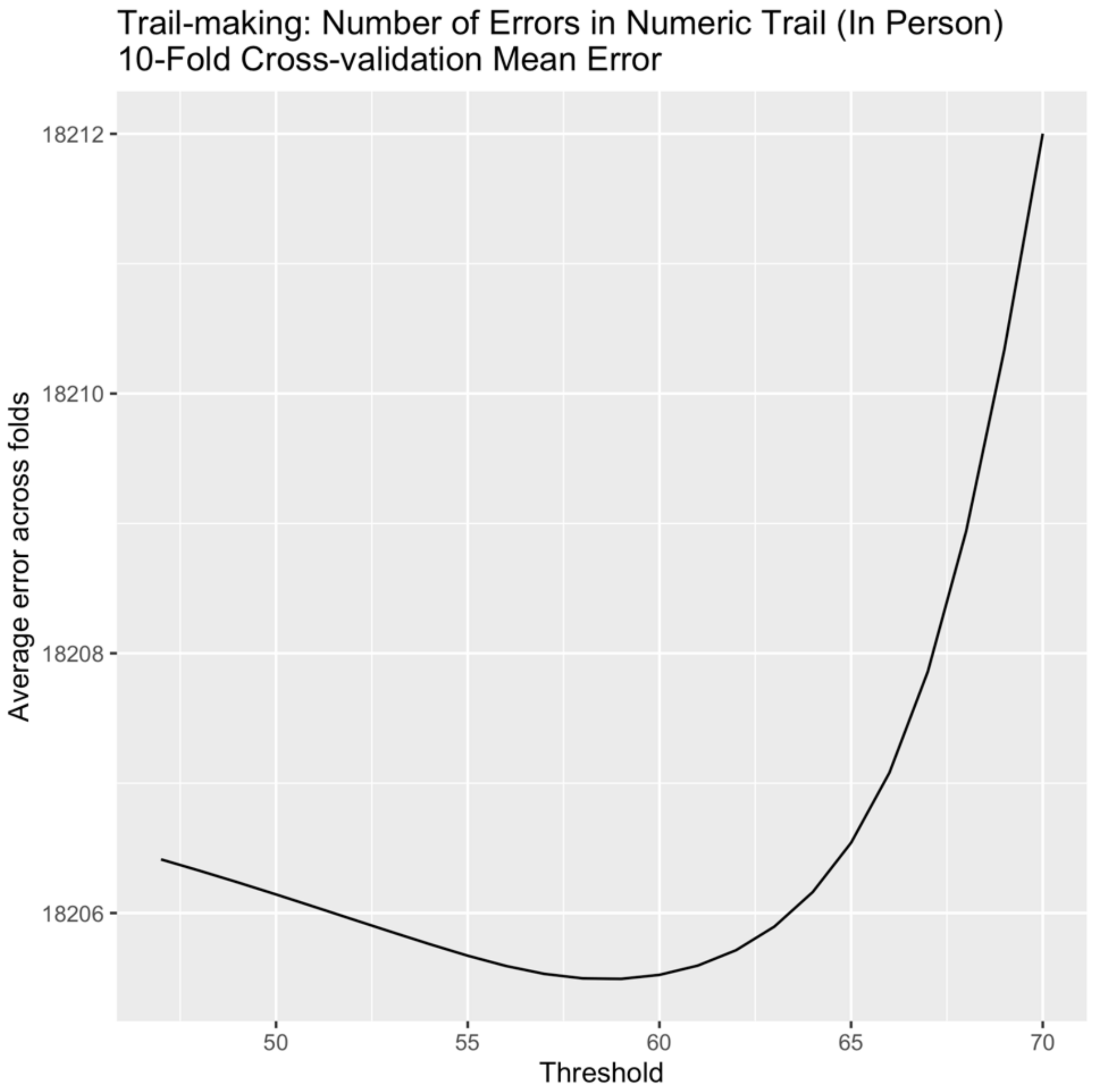

Average CV error is plotted on vertical axis, and the age of divergence for the model ($t_{threshold}$) is on the horizontal axis. The figure shows that the model with divergence in cognitive test score between low and high AD-GRS at age 59 was the best CV fit to the data based on the location of the minimum error.

© 2022 Zimmerman SC et al. *JAMA Network Open.*

eFigure 2: Divergence plots for all cognitive tests – part 1

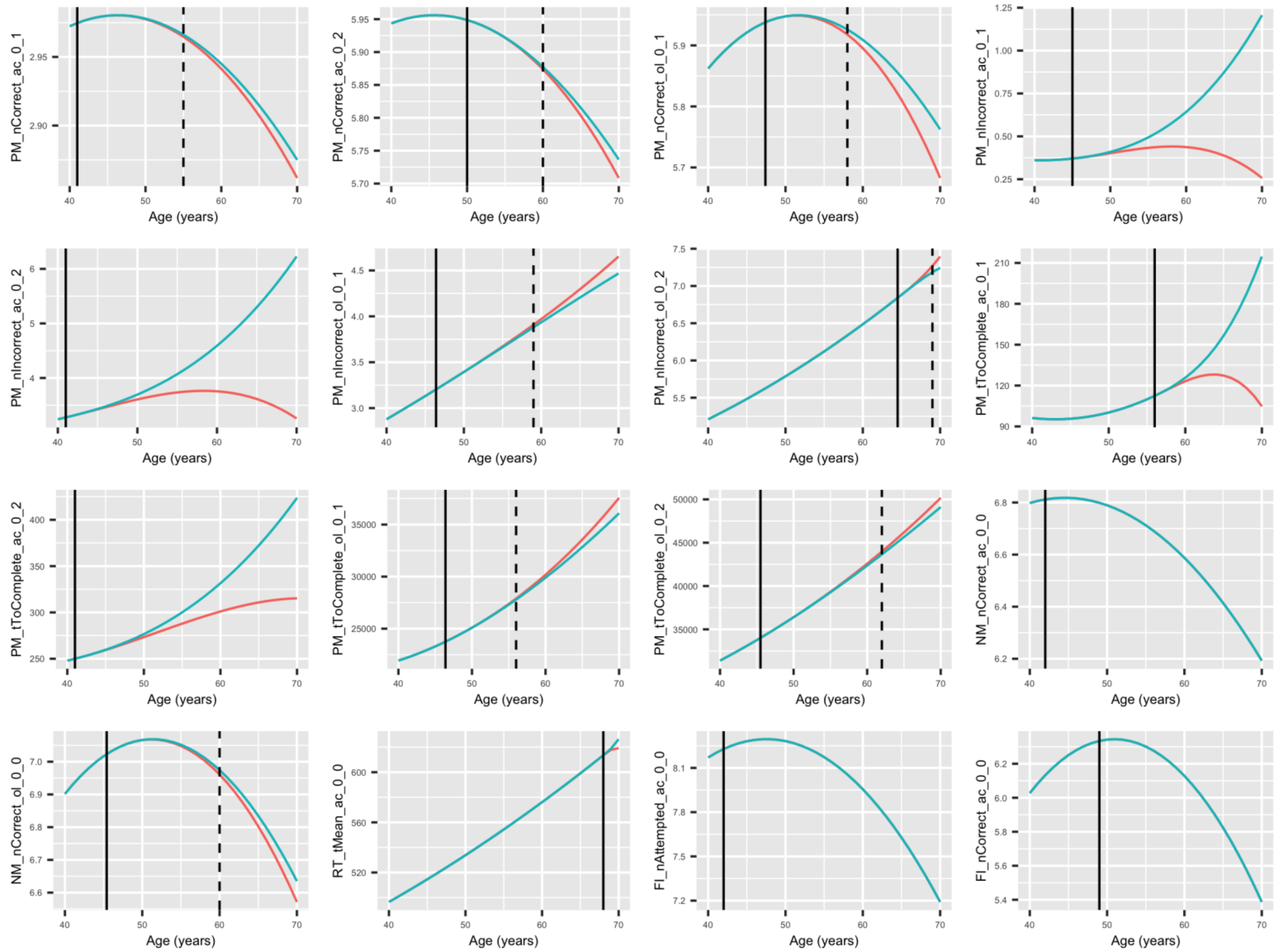

Plots of predicted average cognition of four cognitive measures at higher (1.76) and lower (-1.22) z-scored AD-GRS. Solid vertical lines indicate threshold age at which the AD-GRS begins to influence the cognitive measure. At ages below the threshold, AD-GRS does not interact with age to influence cognition. Dashed vertical lines indicate the earliest age at which the cognitive difference in the

© 2022 Zimmerman SC et al. *JAMA Network Open.*

high and low AD-GRS groups are statistically detectable using a one-sided, two-sample t-test at ($p <= 0.05$). These predictions were based on females with median values of the 10 principal components. Red lines indicate high (95th percentile) AD-GRS and blue lines indicate low(5th percentile) AD-GRS. Key: PM_nCorrect_ac_0_1: 6) Pairs Matching Number Correct (Round 1) In Person. PM_nCorrect_ac_0_2: 7) Pairs Matching Number Correct (Round 2) In Person. PM_nCorrect_ol_0_1: 12) Pairs Matching Number Correct (Round 1) Online. PM_nIncorrect_ac_0_1: 8) Pairs Matching Number Incorrect (Round 1) In Person. PM_nIncorrect_ac_0_2: 9) Pairs Matching Number Incorrect (Round 2) In Person. PM_nIncorrect_ol_0_1: 13) Pairs Matching Number Incorrect (Round 1) Online. PM_nIncorrect_ol_0_2: 14) Pairs Matching Number Incorrect (Round 2) Online. PM_tToComplete_ac_0_1: 10) Pairs Matching Time To Complete (Round 1) In Person. PM_tToComplete_ac_0_2: 11) Pairs Matching Time To Complete (Round 2) In Person. PM_tToComplete_ol_0_1: 15) Pairs Matching Time To Complete (Round 1) Online. PM_tToComplete_ol_0_2: 16) Pairs Matching Time To Complete (Round 2) Online. NM_nCorrect_ac_0_0: 4) Numeric Memory Number Correct In Person. NM_nCorrect_ol_0_0: 5) Numeric Memory Number Correct Online. RT_tMean_ac_0_0: 19) Reaction Time Mean Time In Person. FI_nAttempted_ac_0_0: 2) Fluid Intelligence Number Attempted In Person. FI_nCorrect_ac_0_0: 1) Fluid Intelligence Number Correct In Person.

© 2022 Zimmerman SC et al. *JAMA Network Open.*

eFigure 3: Divergence plots for all cognitive tests – part 2

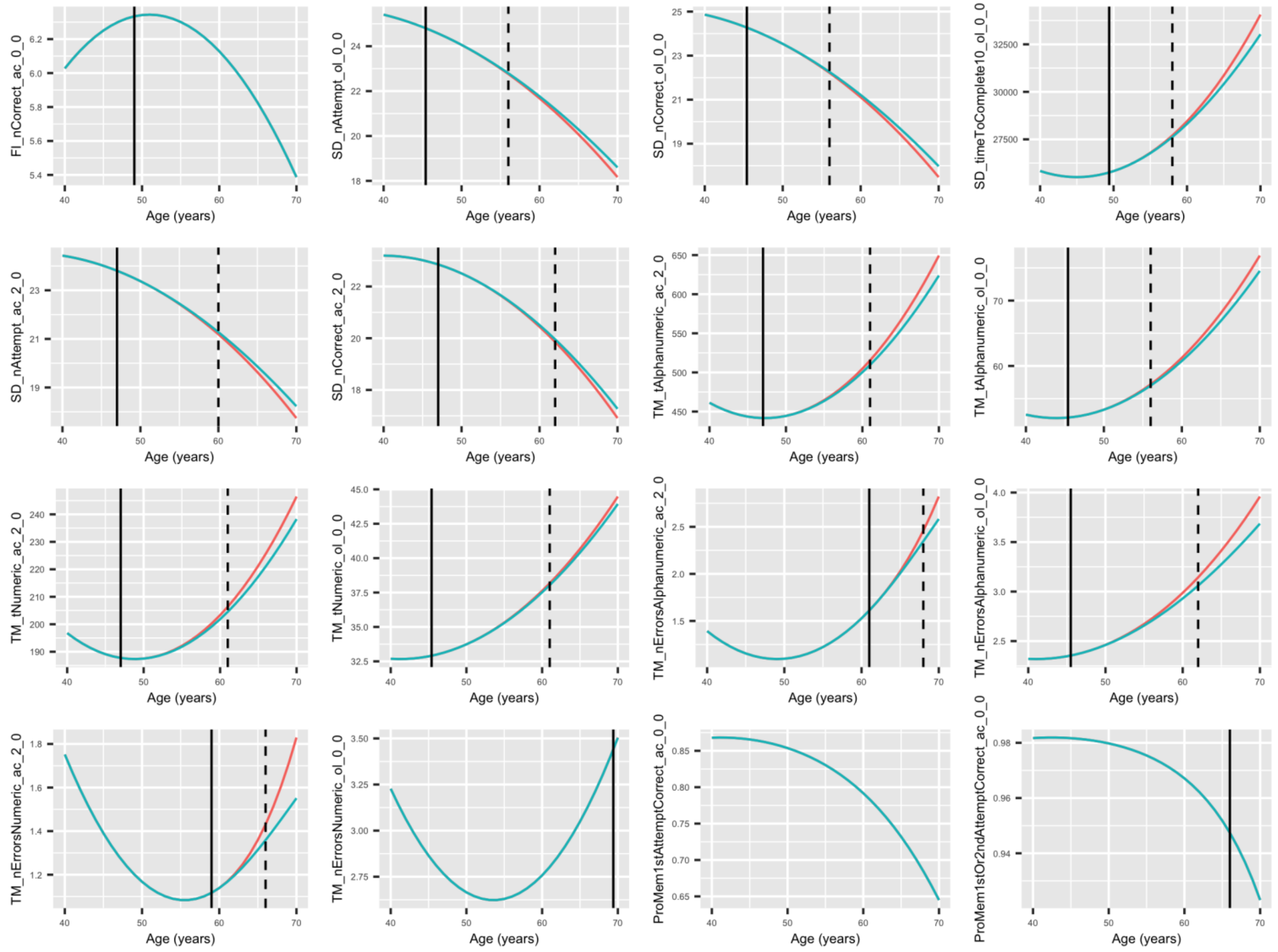

Plots of predicted average cognition of four cognitive measures at higher (1.76) and lower (-1.22) z-scored AD-GRS. Solid vertical lines indicate threshold age at which the AD-GRS begins to influence the cognitive measure. At ages below the threshold, AD-GRS does not interact with age to influence cognition. Dashed vertical lines indicate the earliest age at which the cognitive difference in the high and low AD-GRS groups are statistically detectable using a one-sided, two-sample t-test at ($p <= 0.05$). These predictions were based on females with median values of the 10 principal components. Red lines indicate high (95th percentile) AD-GRS and blue lines

© 2022 Zimmerman SC et al. *JAMA Network Open.*

indicate low(5th percentile) AD-GRS. Key: FI_nCorrect_ol_0_0: 3) Fluid Intelligence Number Correct Online. SD_nAttempt_ol_0_0: 23) Symbol Digit Substitution Number Attempted Online. SD_nCorrect_ol_0_0: 22) Symbol Digit Substitution Number Correct Online. SD_timeToComplete10_ol_0_0: 24) Symbol Digit Substitution Time To Complete 10 Substitutions Online. SD_nAttempt_ac_2_0: 20) Symbol Digit Substitution Number Attempted In Person. SD_nCorrect_ac_2_0: 21) Symbol Digit Substitution Number Correct In Person. TM_tAlphanumeric_ac_2_0: 27) Trail-making Time to Complete Alphanumeric Trail In Person. TM_tAlphanumeric_ol_0_0: 30) Trail-making Time to Complete Alphanumeric Trail Online. TM_tNumeric_ac_2_0: 25) Trail-making Time to Complete Numeric Trail In Person. TM_tNumeric_ol_0_0: 29) Trail-making Time to Complete Numeric Trail Online. TM_nErrorsAlphanumeric_ac_2_0: 28) Trail-making Number Of Errors In Alphanumeric Trail In Person. TM_nErrorsAlphanumeric_ol_0_0: 32) Trail-making Number Of Errors In Alphanumeric Trail Online. TM_nErrorsNumeric_ac_2_0: 26) Trail-making Number Of Errors In Numeric Trail In Person. TM_nErrorsNumeric_ol_0_0: 31) Trail-making Number Of Errors In Numeric Trail Online. ProMem1stAttemptCorrect_ac_0_0: 17) Prospective Memory 1st Attempt Correct In Person. ProMem1stOr2ndAttemptCorrect_ac_0_0: 18) Prospective Memory 1st Or 2nd Attempt Correct In Person.

© 2022 Zimmerman SC et al. *JAMA Network Open.*

eFigure 4: Mean cognitive scores by age and ADGRS, unadjusted for covariates – Part 1

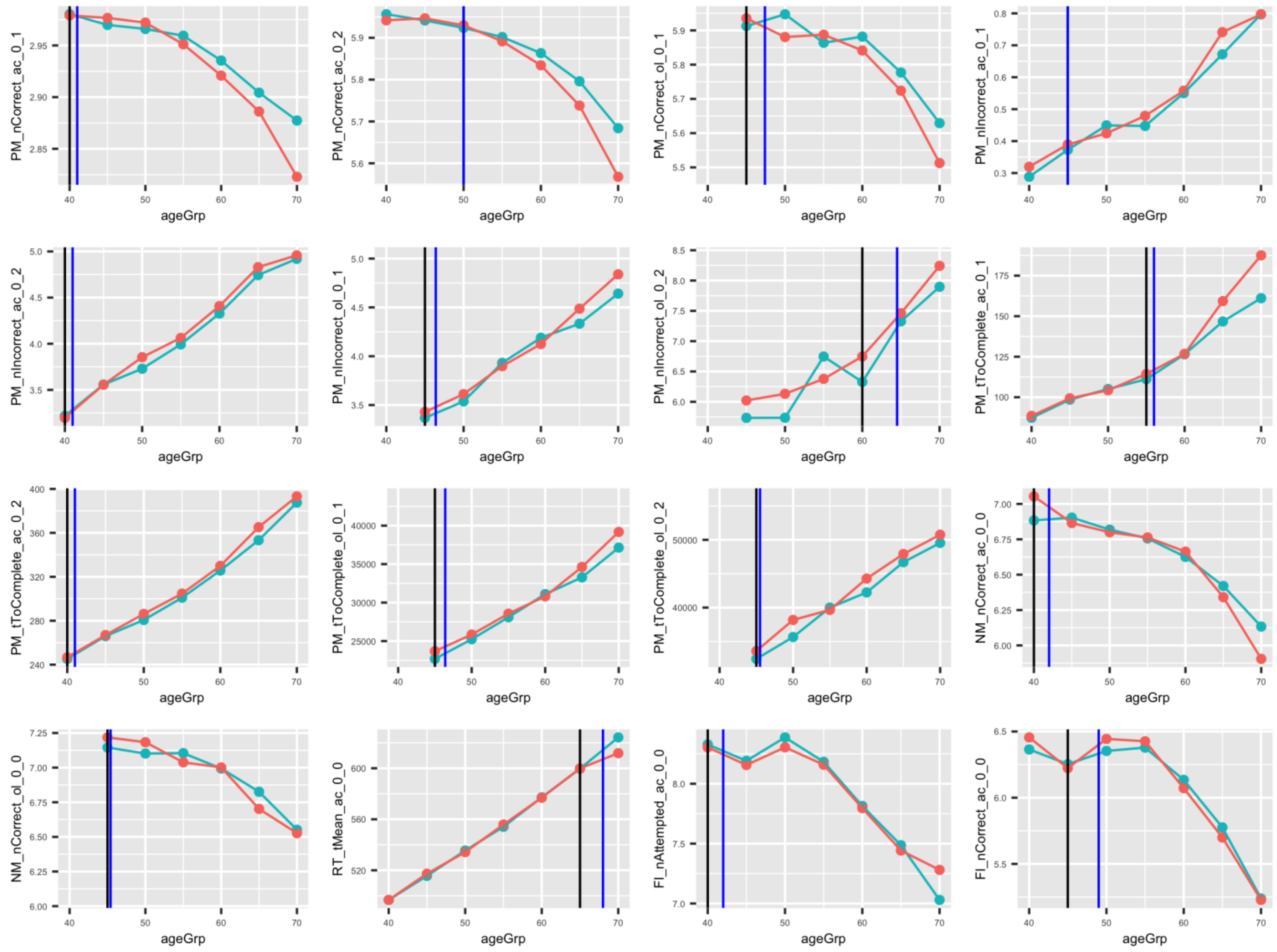

Blue vertical lines indicate 5-year age group at which divergence was detected in the main analysis. Black vertical lines denote the minimum of the 5-year age group containing the blue vertical line. Red series indicates mean cognitive score among participants in the highest quartile of AD-GRS, a blue series indicates mean cognitive score among participants in the lowest quartile of AD-GRS. Key: PM_nCorrect_ac_0_1: 6) Pairs Matching Number Correct (Round 1) In Person. PM_nCorrect_ac_0_2: 7) Pairs Matching Number

© 2022 Zimmerman SC et al. *JAMA Network Open.*

Correct (Round 2) In Person. PM_nCorrect_ol_0_1: 12) Pairs Matching Number Correct (Round 1) Online. PM_nIncorrect_ac_0_1: 8) Pairs Matching Number Incorrect (Round 1) In Person. PM_nIncorrect_ac_0_2: 9) Pairs Matching Number Incorrect (Round 2) In Person. PM_nIncorrect_ol_0_1: 13) Pairs Matching Number Incorrect (Round 1) Online. PM_nIncorrect_ol_0_2: 14) Pairs Matching Number Incorrect (Round 2) Online. PM_tToComplete_ac_0_1: 10) Pairs Matching Time To Complete (Round 1) In Person. PM_tToComplete_ac_0_2: 11) Pairs Matching Time To Complete (Round 2) In Person. PM_tToComplete_ol_0_1: 15) Pairs Matching Time To Complete (Round 1) Online. PM_tToComplete_ol_0_2: 16) Pairs Matching Time To Complete (Round 2) Online. NM_nCorrect_ac_0_0: 4) Numeric Memory Number Correct In Person. NM_nCorrect_ol_0_0: 5) Numeric Memory Number Correct Online. RT_tMean_ac_0_0: 19) Reaction Time Mean Time In Person. FI_nAttempted_ac_0_0: 2) Fluid Intelligence Number Attempted In Person. FI_nCorrect_ac_0_0: 1) Fluid Intelligence Number Correct In Person.

eFigure 5: Mean cognitive scores by age and ADGRS, unadjusted for covariates – Part 2

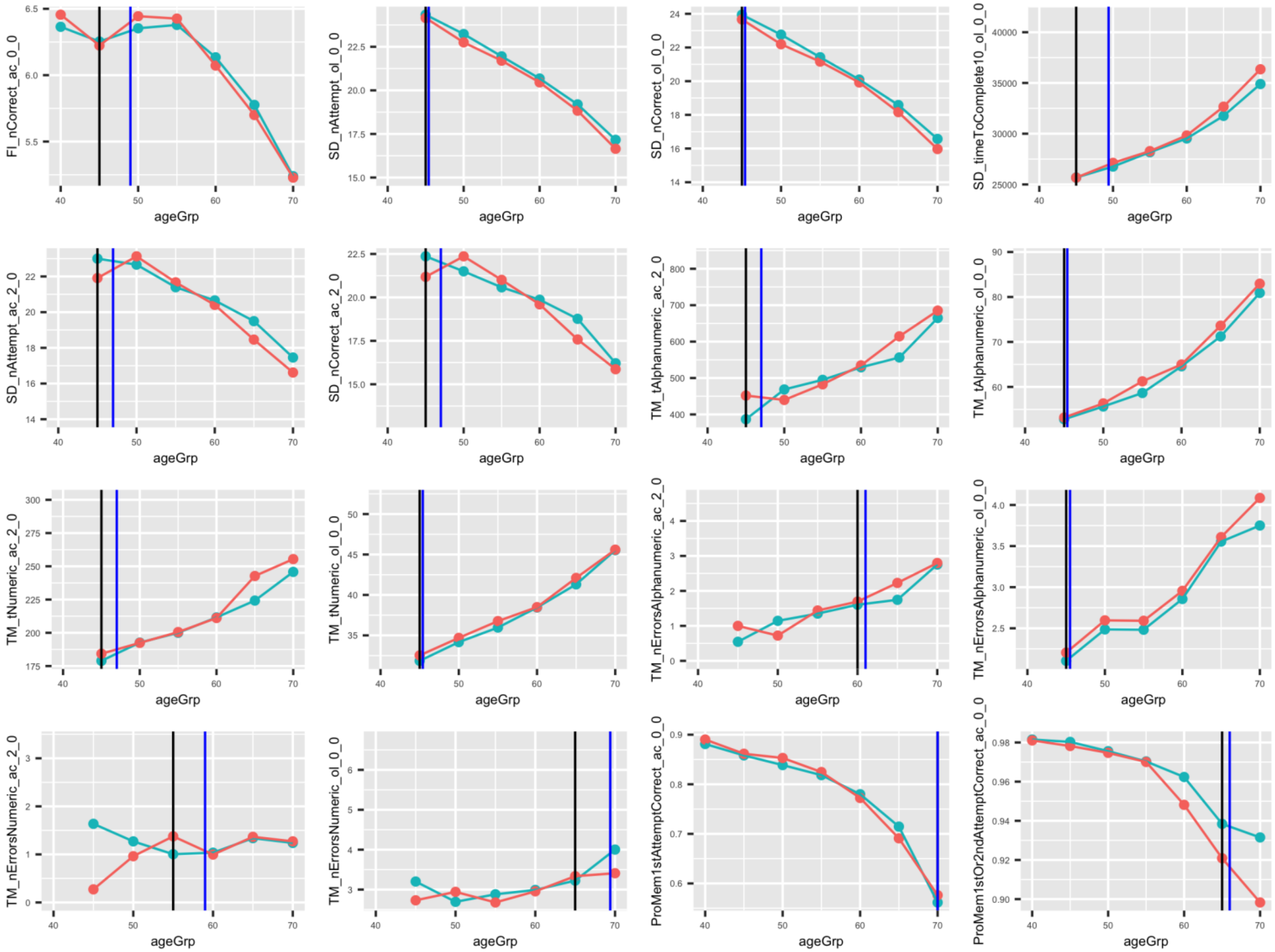

Blue vertical lines indicate 5-year age group at which divergence was detected in the main analysis. Black vertical lines denote the minimum of the 5-year age group containing the blue vertical line. Red series indicates mean cognitive score among participants in the highest quartile of AD-GRS, a blue series indicates mean cognitive score among participants in the lowest quartile of AD-GRS. Key: FI_nCorrect_ol_0_0: 3) Fluid Intelligence Number Correct Online. SD_nAttempt_ol_0_0: 23) Symbol Digit Substitution Number Attempted Online. SD_nCorrect_ol_0_0: 22) Symbol Digit Substitution Number Correct Online. SD_timeToComplete10_ol_0_0: 24)

© 2022 Zimmerman SC et al. *JAMA Network Open.*

Symbol Digit Substitution Time To Complete 10 Substitutions Online. SD_nAttempt_ac_2_0: 20) Symbol Digit Substitution Number Attempted In Person. SD_nCorrect_ac_2_0: 21) Symbol Digit Substitution Number Correct In Person. TM_tAlphanumeric_ac_2_0: 27) Trail-making Time to Complete Alphanumeric Trail In Person. TM_tAlphanumeric_ol_0_0: 30) Trail-making Time to Complete Alphanumeric Trail Online. TM_tNumeric_ac_2_0: 25) Trail-making Time to Complete Numeric Trail In Person. TM_tNumeric_ol_0_0: 29) Trail-making Time to Complete Numeric Trail Online. TM_nErrorsAlphanumeric_ac_2_0: 28) Trail-making Number Of Errors In Alphanumeric Trail In Person. TM_nErrorsAlphanumeric_ol_0_0: 32) Trail-making Number Of Errors In Alphanumeric Trail Online. TM_nErrorsNumeric_ac_2_0: 26) Trail-making Number Of Errors In Numeric Trail In Person. TM_nErrorsNumeric_ol_0_0: 31) Trail-making Number Of Errors In Numeric Trail Online. ProMem1stAttemptCorrect_ac_0_0: 17) Prospective Memory 1st Attempt Correct In Person. ProMem1stOr2ndAttemptCorrect_ac_0_0: 18) Prospective Memory 1st Or 2nd Attempt Correct In Person.

© 2022 Zimmerman SC et al. *JAMA Network Open.*

eReferences

1. Cognitive function - Online follow-up. UK Biobank Data Showcase. Accessed June 24, 2021. https://biobank.ndph.ox.ac.uk/showcase/label.cgi?id=116

2. Cognitive function - UK Biobank Assessment center. UK Biobank Data Showcase. Accessed June 24, 2021. https://biobank.ndph.ox.ac.uk/showcase/label.cgi?id=100026

3. Lambert J-C, Ibrahim-Verbaas CA, Harold D, et al. Meta-analysis of 74,046 individuals identifies 11 new susceptibility loci for Alzheimer's disease. *Nature Genetics*. 2013;45(12):1452-1458. doi:10.1038/ng.2802

© 2022 Zimmerman SC et al. *JAMA Network Open.*
